# PCSK9 facilitates melanoma pathogenesis via a network regulating tumor immunity

**DOI:** 10.1186/s13046-022-02584-y

**Published:** 2023-01-02

**Authors:** Yan Gu, Xiaozeng Lin, Ying Dong, Geoffrey Wood, Nabil G. Seidah, Geoff Werstuck, Pierre Major, Michael Bonert, Anil Kapoor, Damu Tang

**Affiliations:** 1 Urological Cancer Center for Research and Innovation (UCCRI), T3310, St. Joseph’s Hospital, 50 Charlton Ave East, Hamilton, ON L8N 4A6 Canada; 2 Department of Surgery, McMaster University, Hamilton, ON L8S 4K1 Canada; 3 The Research Institute of St Joe’s Hamilton, G344, St. Joseph’s Hospital, Hamilton, ON L8N 4A6 Canada; 4 Department of Pathology, University of Guelph, Guelph, ON N1G 2W1 Canada; 5 Laboratory of Biochemical Neuroendocrinology, Montreal Clinical Research Institute, University of Montreal, Montreal, QC H2W 1R7 Canada; 6 Department of Medicine, McMaster University, Hamilton, ON L8S 4K1 Canada; 7 Department of Oncology, McMaster University, Hamilton, ON L8S 4K1 Canada; 8 Department of Pathology and Molecular Medicine, McMaster University, Hamilton, ON L8S 4K1 Canada

**Keywords:** PCSK9, Melanoma, Tumorigenesis, Immune checkpoint blockade (ICB), Biomarkers of ICB

## Abstract

**Background:**

PCSK9 regulates cholesterol homeostasis and promotes tumorigenesis. However, the relevance of these two actions and the mechanisms underlying PCSK9’s oncogenic roles in melanoma and other cancers remain unclear.

**Methods:**

PCSK9’s association with melanoma was analysed using the TCGA dataset. Empty vector (EV), PCSK9, gain-of-function (D374Y), and loss-of-function (Q152H) PCSK9 mutant were stably-expressed in murine melanoma B16 cells and studied for impact on B16 cell-derived oncogenesis in vitro and in vivo using syngeneic C57BL/6 and *Pcsk9*^*−/−*^ mice. Intratumoral accumulation of cholesterol was determined. RNA-seq was performed on individual tumor types. Differentially-expressed genes (DEGs) were derived from the comparisons of B16 PCSK9, B16 D374Y, or B16 Q152H tumors to B16 EV allografts and analysed for pathway alterations.

**Results:**

PCSK9 expression and its network negatively correlated with the survival probability of patients with melanoma. PCSK9 promoted B16 cell proliferation, migration, and growth in soft agar in vitro, formation of tumors in C57BL/6 mice in vivo, and accumulation of intratumoral cholesterol in a manner reflecting its regulation of the low-density lipoprotein receptor (LDLR): Q152H, EV, PCSK9, and D374Y. Tumor-associated T cells, CD8 + T cells, and NK cells were significantly increased in D374Y tumors along with upregulations of multiple immune checkpoints, IFNγ, and 143 genes associated with T cell dysfunction. Overlap of 36 genes between the D374Y DEGs and the PCSK9 DEGs predicted poor prognosis of melanoma and resistance to immune checkpoint blockade (ICB) therapy. CYTH4, DENND1C, AOAH, TBC1D10C, EPSTI1, GIMAP7, and FASL (FAS ligand) were novel predictors of ICB therapy and displayed high level of correlations with multiple immune checkpoints in melanoma and across 30 human cancers. We observed FAS ligand being among the most robust biomarkers of ICB treatment and constructed two novel and effective multigene panels predicting response to ICB therapy. The profiles of allografts produced by B16 EV, PCSK9, D374Y, and Q152H remained comparable in C57BL/6 and *Pcsk9*^*−/−*^ mice.

**Conclusions:**

Tumor-derived PCSK9 plays a critical role in melanoma pathogenesis. PCSK9’s oncogenic actions are associated with intratumoral cholesterol accumulation. PCSK9 systemically affects the immune system, contributing to melanoma immune evasion. Novel biomarkers derived from the PCSK9-network effectively predicted ICB therapy responses.

**Supplementary Information:**

The online version contains supplementary material available at 10.1186/s13046-022-02584-y.

## Background

Melanoma represents 2% of skin cancer cases. However, it accounts for approximately 80% of skin cancer deaths [1]. As the most lethal form of skin cancer, melanoma shows high proliferative potential and significant risk to metastasize to brain and other organs. The rapid growth rate is partially attributable to the activation of the RAS-RAF-MEK-ERK pathway, a well-established signaling process promoting cell cycle progression [2]. Mutations leading to activation of BRAF (*BRAF*^*V600*^ mutations) and NRAS occur in 50% [3] and 15–20% [4] of melanoma respectively. Approximately 80% of melanomas contain either *BRAF* or *NRAS* activating mutations [5, 6]. Mutations responsible for activation of oncogenes and inactivation of tumor suppressors (*NF1*, *CDKN2A*, *p53*, and *PTEN*) are typical features of melanoma [7], which reflects melanoma carrying the highest mutational load across all human cancer spectrum [8]. The features of prevalent activating mutations in *BRAF* and the high mutational burden in melanoma have been explored for targeted therapy with the combination of BRAF and MEK inhibitors [9] and immunotherapy involving immune checkpoint blockade (ICB), anti-CTLA-4 (cytotoxic T-lymphocyte antigen-4) antibody (ipilimumab) and anti-PD-1 (programmed cell death 1) antibodies (nivolumab and pembrolizumab) [10]. The approval of these ICB by the FDA in 2011 (ipilimumab) and 2014 (nivolumab and pembrolizumab) transformed the management of metastatic melanoma, which extends long-term survival rate from less than 10% to 4-year survival rate exceeding 50% [10, 11]. Nonetheless, for the large number of patients who either do not respond or acquire resistance to ICB, our knowledge of the mechanisms and factors underpinning this resistance remains limited [10, 12].

Abnormalities in lipid metabolism are a contributing factor to the rapid growth and alteration in immune profiles of melanoma. Increases in cell proliferation require coordination with lipid availability, including fatty acids and cholesterol. Cancer cells require elevations in de novo lipogenesis via utilization of exogenous fatty acids to support their rapid proliferation [13]; cholesterol constitutes 30% of the cell membrane and is essential for cell proliferation [14]. Additionally, cholesterol contributes to signalling pathways promoting tumorigenesis and progression via the formation of cholesterol-enriched membrane microdomains [15, 16] and covalent modifications of component proteins required for hedgehog signalling [17, 18]. Accumulative evidence supports increases in cholesterol uptake and biosynthesis as a typical feature of cancer [19]. Cholesterol and its metabolites are major contributors to immunosuppressive tumor microenvironment (TME) [19]. In melanoma, an increase in cholesterol biosynthesis is correlated with poor prognosis [20].

Proprotein convertase subtilisin kexin type 9 (PCSK9) is a major regulator of cholesterol homeostasis via binding and downregulating low-density lipoprotein receptor (LDLR) [21–25]. PCSK9 displays oncogenic actions in cancer [26], including gastric cancer [27], esophageal cancer [28], prostate cancer [29–31], breast cancer [32], ovarian cancer [33], lung cancer [34–37], hepatocellular carcinoma [38, 39], and melanoma [40, 41]. While accumulative evidence reveals a general action of PCSK9 in facilitating tumorigenesis, the specific association of PCSK9’s role in regulating lipid metabolism and its oncogenic potential remains unclear.

Nonetheless, recent evidence supports a relationship between PCSK9 and ICB-based immunotherapy. In hepatocellular carcinoma, PCSK9 enhances M2 polarization of tumor-associated macrophages (TAMs) [39]; M2 macrophages contribute to cancer progression [42]. In patients with advanced non-small cell lung cancer (NSCLC), low levels of baseline serum PCSK9 were associated with increases in overall survival (OS) [35, 36]. In both syngeneic mouse models for colon cancer and melanoma, anti-PD1 therapy displayed synergy with anti-PCSK9 antibody in inhibition of tumor growth [43]. Analogous to PCSK9’s action of downregulating LDLR, PCSK9 instigates MHC I reduction from cancer cell surface likely via lysosome-based degradation, in a manner that is independent of LDLR regulation [43]. However, PCSK9 also downregulates LDLR on CD8 + T cell surface to prevent LDLR-facilitated recycling of T-cell receptor (TCR) [44]. Regardless of PCSK9’s LDLR-dependent and -independent actions, it remains unclear whether PCSK9’s actions in regulating cholesterol homeostasis are relevant to its oncogenic properties.

Considering the strong evidence for PCSK9’s oncogenic involvement, it is surprising that PCSK9 vaccine has no impact on melanoma and only modestly reduces breast cancer growth in murine models [32, 40]. This may be related to the relative inefficiencies of the reported vaccines to completely block PCSK9 activity. In clinical trials on cardiovascular conditions, anti-PCSK9 antibodies evolocumab and alirocumab, which only block the activity of circulating PCSK9 primarily secreted from liver, did not reduce cancer incidence [45, 46], suggesting a much more complex narrative for PCSK9’s oncogenic actions that remains to be uncovered.

We report here a major role of tumor-derived PCSK9 in facilitating the growth of B16 cell-generated allografts in comparison to host-derived PCSK9. This may shed light on the ineffectiveness of the PCSK9 vaccine in mice and of targeting circulating PCSK9 in cancer patients. PCSK9-derived oncogenesis in melanoma is closely associated with lipid accumulation. The gain-of-function (GOF) PCSK9 natural variant D374Y [47] enhanced B16 cell-derived tumor growth compared to PCSK9 along with elevations of lipid accumulation; both events were significantly hindered by the loss-of-function (LOF) mutant Q152H [48]. Differentially expressed genes (DEGs) relevant to the GOF mutant D374Y dominantly affected the immune system. The common DEGs shared by PCSK9 and D374Y tumors stratify poor OS of skin cutaneous melanoma (SKCM) and worse outcome in melanoma as well as other cancers treated with ICB. Additionally, several novel and effective biomarkers in predicting ICB response were identified. Collectively, our research illustrates multiple novel aspects of PCSK9’s oncogenic actions.

## Methods

### Cell lines, plasmid, and retrovirus infection

B16 and 293 T cells were cultured in DMEM cell culture media (Gibco, Carlsbad, CA), supplemented with 1% Penicillin–Streptomycin (Gibco, Carlsbad, CA) and 10% fetal bovine serum (Life Technologies, Burlington, ON). Cell lines were routinely checked for Mycoplasma contamination using a PCR kit (Abm, Cat#: G238). PCSK9, D374Y, and Q152H cDNA plasmid were produced by Dr. Seidah and subcloned into pLNCX-geneticin retroviral plasmid and subsequent transfection was performed following our published conditions [49].

### Wound healing, colony formation, and soft agar assay

B16 EV, B16 Q152H, B16 PCSK9, and B16 D374Y cells (1 × 10^4^) were seeded in 6-well plates and incubated overnight at 37 °C. Each well was scratched using a sterile pipette tip in multiple horizontal stripes to generate the wound. The cells were washed with PBS to remove any dislodged cells and incubated at 37 °C overnight. The plates were examined at 0 h, 6 h, 12 h and 24 h to observe the migration of cells across the wound using a light microscope (Axiovert 200; Carl Zeiss, Jena, Germany)*.* Colony formation assay was conducted by seeding cells in six-well plates with 100, 500, 1000 cells. Cells were cultured for one week in 37 °C and 5% CO_2_ environment. Colonies were fixed by fixation buffer (2% formaldehyde) and stained by crystal violet (0.5%). Crystal violet staining was released with 10% acetic acid and absorbance is measured at 590 nm with spectrometer. Colony numbers were counted and analyzed. Soft agar assay was performed by creating a bottom agar layer in six-well plate using 0.6% agarose prepared in 1.5 mL of 2X DMEM media. While the bottom agar layer solidifies, B16 were dissociated into a single-cell suspension and counted. 25 μl of 10^4^ B16 cells suspension in DMEM/10% FBS were mixed with 1.5 mL of 2X DMEM media containing 20% FBS with 0.6% agar. The mixture was placed on top of the bottom agar layer and allowed to solidify. The plates were incubated with 100 µl of DMEM media containing 10% FBS per well at 37 °C and 5% CO_2_. The media were changed every 4 days. At 30 days, images of each well were taken, and colony numbers were counted and analyzed.

### Western blot

Cell lysates were prepared in buffer containing 20 mM Tris (pH 7.4), 150 mM NaCl, 1 mM EDTA, 1 mM EGTA, 1% Triton X-100, 25 mM sodium pyrophosphate, 1 mM NaF, 1 mM β-glycerophosphate, 0.1 mM sodium orthovanadate, 1 mM PMSF, 2 μg/ml leupeptin and 10 μg/ml aprotinin (Sigma Aldrich). Western blot was carried out as we have previously published [49]. The primary and secondary antibodies used were: anti-PCSK9 (1:1000; Abcam, CA, USA); anti-actin (1:1,000; Santa Cruz Biotechnology), anti-mouse (1:3,000; GE Healthcare) and anti-rabbit (1:3,000).

### Immunofluorescence staining

Deparaffinization of paraffin embedded slides was carried out in xylene, followed by ethanol clearance and antigen retrieval by heat treatment in a sodium citrate buffer (pH = 6.0). Permeabilization was performed with 1X PBS/gelatin (0.2% *w/v*)/Triton (0.25% *v/v*) solution for 10 min twice. Non-specific binding sites were blocked with PBS containing 1% BSA and 10% normal donkey serum (Vector Laboratories) for 1 h, followed by addition of primary antibodies: CD8-alpha (Cell Signaling, 1:100) overnight at 4 °C. Following 3 × 10 min washes with PBS, slides were incubated with secondary antibody FITC Donkey anti-rabbit IgG (Cell Signaling, 1:200), and then in 10 mM CUSO_4_/50 mM NH_4_Cl solution for 10 min. Slides were mounted with VECTASHIELD anti-fade mounting medium with DAPI (Vector Laboratories). Images were captured in 24 h with a fluorescence microscope (Axiovert 200; Carl Zeiss). Quantification of staining was performed using Image J and obtained as % of CD8 + cells normalized to DAPI signal. All area of tumor sections were analyzed (5 tumors/group) and average number of cells per tumor section used for quantification were approximately 9.8 × 10^3^.

### Filipin and ORO staining

Frozen sections were fixed with 4% paraformaldehyde for 20 min and permeabilized with 0.05% Triton X-100 for 15 min. Following 3 × PBS washes, the slides were quenched with 1.5 mg/mL glycine in PBS for 10 min and stained with filipin (50 μg/mL, Sigma) for 2 h in the dark. After washing with PBS, the slide was covered with VECTASHIELD anti-fade mounting medium (Vector Laboratories). Images were captured immediately with a fluorescence microscope (Axiovert 200; Carl Zeiss). For Oil-Red-O (ORO) staining, frozen slides were warmed to room temperature and incubated in 10% formalin for 1 h. Slides were serially washed with distilled water and 60% isopropanol. ORO working solution (Sigma) was prepared according to manufacturer’s instruction and filtered twice through 45-μm filter to remove precipitates. Slides were incubated with ORO working solution at RT for 15 min, counterstained with Mayer’s hematoxylin (Sigma) for 15 s and rinsed under running tap water. Slides were mounted with aqueous mounting media and images were taken with a light microscope. Quantification of staining were performed with Axiovision and quantity of red pixels in relation to μm^2^ of section area were calculated.

### Allograft tumor formation

B16 EV, B16 Q152H, B16 PCSK9, and B16 D374Y cells (1 × 10^5^/per mice) were counted and suspended in 0.1 ml DMEM/Matrigel (BD) mixture with 1:1 volume and implanted subcutaneously into the left flank of 8-week-old C57BL/6 (The Jackson Laboratory) or *Pcsk9*^*−/−*^ (Dr. Seidah) male mice. After implantation of cancer cells, the mice were monitored through observation and palpation. The size of the tumors was measured every two days by caliper. Tumor volume was calculated based on the formula V = L × W^2^ × 0.52. Animals were euthanized when the tumor volume reached endpoint (tumor size > 1000 mm^3^ or poor body conditions). The allograft tumors were collected and processed for subsequent analysis.

### Quantitative real-time PCR

Total RNA was isolated from allograft tissues of B16 EV, B16 Q152H, B16 PCSK9, and B16 D374Y with the Iso-RNA Lysis Reagent (5 PRIME); reverse transcription was performed using Superscript III (Thermo Fisher Scientific). Quantitative real-time PCR was performed using the ABI 7500 Fast Real-Time PCR System (Applied Biosystems, Foster, California, USA) using SYBR-green (Thermo Fisher Scientific). A detailed list for the primers sequence is organized (Additional file 20: Table S1). Fold changes were calculated using the formula: 2^−ΔΔCt^.

### RNA sequencing analysis

RNA sequencing analysis was carried out following our established conditions [50]. RNA was extracted from B16 EV, B16 Q152H, B16 PCSK9, and B16 D374Y allografts (*n* = 3 per group) using a miRNeasy Mini Kit (Qiagen, No. 217004). RNA was enriched for poly(A) mRNA using NEBNext® Poly(A) mRNA Magnetic Isolation Modules. Unique dual indexes were used for library preparation. The libraries were sequenced by McMaster Genomics Facility using a pair-end, 2 × 50 bp configuration on an Illumina NextSeq 2000 P3 flow cell, with 50 M clusters aimed per sample. RNA-seq reads were processed and analyzed using Galaxy (https://usegalaxy.org/). Low quality reads and adaptor sequences were removed. Alignment to mouse genomic sequence (mm10) was carried out using HISAT2; read counts were then performed with the “Featurecounts” function. Differential gene expression was determined using DESeq2. KEGG analysis and GSEA (Gene Set Enrichment Analysis) were performed using Galaxy; the FGSEA (fast preranked GSEA) was used for GSEA analysis. Enrichment analyses were carried out using Metascape [51] (https://metascape.org/gp/index.html#/main/step1).

### Quantification of tumor-associated immune cells

RNA-seq reads were first processed by removing adaptor sequences and low-quality reads. Reads generated by “Featurecounts” following alignment to mouse genomic sequence version 10 (mm10) were quantified by FPKM (fragment per kilobase million) using the R package countToFPKM, which were then converted to TPM (transcripts per million) quantification with the R RNAontheBENCH package. Immune cell contents associated with B16 tumors were determined using the mouse MCP (mMCP) computation program (R mMCPcounter package) [52]. CD8 + T cell infiltration was analyzed using immunofluorescence staining (see Immunofluorescence staining section for details).

### Variable selection and regression analysis

The TCGA PanCancer Atlas SKCM dataset available from cBioPortal [53, 54] was used to construct a *n* = 13 multigene signature (SigPCSK9NW) from the PCSK9 network (NW). The detailed strategy involved in this construction was presented in Additional file 1: Fig S1. Briefly, A cutoff point was first defined to stratify tumors in the TCGA cohort into a high- and low-risk of mortality group at *p* < 0.01 (logrank test). A set of differentially expressed genes (DEGs) were then obtained in this setting; a cohort with DEG expressions and the relevant clinical data was subsequently retrieved from the TCGA dataset. Random division of the cohort into a training and testing sub-population at 6:4 was performed using R. The training population was used to select variables for predicting mortality risk using the Cox-based Elastic-net program within the R glnmet package with the α mixing parameter set at 0.2. Three rounds of selection were performed; all unique genes (*n* = 23) were combined into a multigene panel; the size of this panel was subsequently optimized with the BeSS R package, leading to SigPCSK9NW consisting of *n* = 13 component genes.

Cox proportional hazards (Cox PH) regression analyses were performed using the R *survival* package. The PH assumption was tested. Cutoff points were estimated using Maximally Selected Rank Statistics (the *Maxstat* package) in R.

### Correlation analysis of gene expression with immune checkpoints

The TISIDB (tumor-immune system interactions and drug bank database) platform (http://cis.hku.hk/TISIDB/index.php) [55] has organized *n* = 24 immune checkpoint expressions across *n* = 30 TCGA cancer types. The platform enables analysis of correlations of design gene expressions with these immune checkpoint expressions in individual cancer types and across all 30 cancer types. We have input our gene of interest into the TISIDB platform and analyze Spearman correlations of our genes with these immune checkpoint expressions in melanoma and all 30 cancer types.

### Analysis of individual genes and multigene panels for their biomarker values in assessing response to immune checkpoint blockade therapy

We used an elegantly developed new platform TIDE (Tumor Immune Dysfunction and Exclusion) [56, 57] to analyze our genes’ biomarker potential of immune checkpoint blockade (ICB) therapy. TIDE contains two functional applications: Regulator Prioritization and Biomarker Evaluation.

Regulator Prioritization presents 5 core datasets covering neuroblastoma (E-MTAB-179), leukemia (GSE12417_GPl570), breast cancer (METABRIC), endometrial cancer, and melanoma for analyzing factors for association with T cell dysfunction. We used this function to analyze multigene panels for association with T cell dysfunction and screened all D374Y DEGs to identify those with positive correlation with T cell disfunction in all five datasets. These analyses were performed using the human genes in correspondence of the mouse DEGs. A total of *n* = 143 genes were identified (see Additional file 27: Table S8) and were analyzed for their biomarker potential of ICB therapy using the Biomarker Evaluation function.

Biomarker Evaluation allows analysis of individual genes and multigene panels for biomarker values in predicting resistance to ICB therapy in 25 well-organized cancer datasets along with comparisons against a set of well-studied and powerful ICB biomarkers up to date. The details of these 25 datasets are presented in Supplementary materials. We input our genes (individually or as multigene panels) into the Biomarker Evaluation function within TIDE to obtain their ICB biomarker values. The outputs of biomarker values as AUC (area under the curve) for our genes and the ICB biomarkers provided by TIDE were then compared for predicting response to ICB therapy across all 25 cohorts.

### Examination of gene expression

Gene expressions were determined using the UALCAN platform (ualcan.path.uab.edu/home) [58]. Briefly, individual genes were input into the program; their expressions in the setting of primary and metastasis skin melanoma in the TCGA SKMC dataset were then analyzed. Statistical analysis and boxplots were retrieved from the platform.

### Statistical analysis

Kaplan–Meier survival analyses and logrank test were conducted by R *Survival* package and tools provided by cBioPortal. Cox regression analyses were performed using R *survival* package. Time-dependent receiver operating characteristic (tROC) analyses were carried out with R *timeROC* package. ROC and precision-recall (PR) profiles were constructed using the PRROC package in R. Two-tailed Student t-test, one-way ANOVA and two-way ANOVA were performed for statistical analysis of two or more groups comparison respectively, with *p* < 0.05 to be considered statistically significant. Tukey’s test was performed for post-hoc analysis. Statistical analysis was conducted by GraphPad Prism 7 and SPSS 26. Data were presented as mean ± SEM/SD. A value of *p* < 0.05 was considered statistically significant.

## Results

### PCSK9 network robustly predicts the risk of poor prognosis of skin cutaneous melanoma

**Fig. 1:**
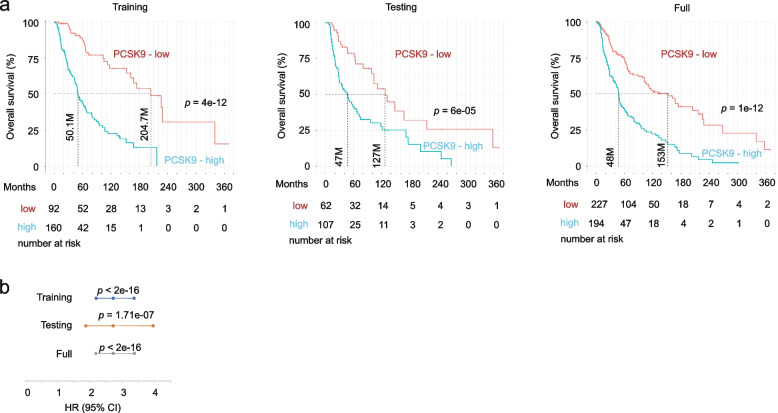
Stratification of overall survival (OS) probabilities of patients with SKMC. Risk score of SigPCSK9NW was produced with Cox regression and cutoff point was defined using Maximally Selected Rank Statistics. The stratification of the risk score on OS of patients with SKMC in the training, testing, and intact cohort (TCGA PanCancer Atlas SKMC) is presented (**a**); the risk score in predicting the risk of poor OS was also analyzed (**b**). Logrank test was performed

Increases in PCSK9 mRNA expression are associated with an elevated risk of poor prognosis in SKCM [43]. To further analyze this association, we determined the potential of PCSK9 network (NW) in predicting poor OS of SKCM. With the cut-off point set at 1.5 z score, PCSK9 mRNA expression separates tumors into two groups with low- and high-risk fatality (Additional file 1: Fig. S1a). A set of DEGs (*n* = 2141 defined as q < 0.05 and fold change ≥|1.5|) between both groups were derived (Additional file 1: Fig. S1a; Additional file 21: Table S2a). DEG expression and the relevant clinical data were retrieved from the TCGA PanCancer Atlas SKCM population within cBioPortal; the cohort was randomly divided into a training and testing population at 6:4 ratio (Additional file 1: Fig. S1a). Using the training population, a multigene panel was constructed for predicting poor OS with Elastic-net logistic regression within the R glmnet package; the gene panel (*n* = 23) was then optimized using the R BeSS program, resulting in a 13-gene signature (SigPCSK9NW) (Additional file 1: Fig. S1a; Additional file 21: Table S2b). SigPCSK9NW effectively stratifies poor OS in the training, testing, and full SKCM cohort (Fig. 1a) and predicts the fatality risk (Fig. 1b). In both the training and testing sub-populations, SigPCSK9NW discriminates melanoma death with comparable ROC AUC (receiver-operating characteristic area under the curve) and PR (precision-recall) AUC curves (Additional file 1: Fig S1b, 1c). SigPCSK9NW score correlates with PCSK9 expression (Additional file 1: Fig S1d), supporting SigPCSK9NW being derived from the PCSK9 network. However, as SigPCSK9NW is substantially more robust than PCSK9 in the stratification of poor OS (comparing Fig. 1a to Additional file 1: Fig. S1a), this suggests that a broader PCSK9 network may be critical in executing its oncogenic functions.

### PCSK9 enhances melanoma growth along with increases in lipid accumulation

**Fig. 2:**
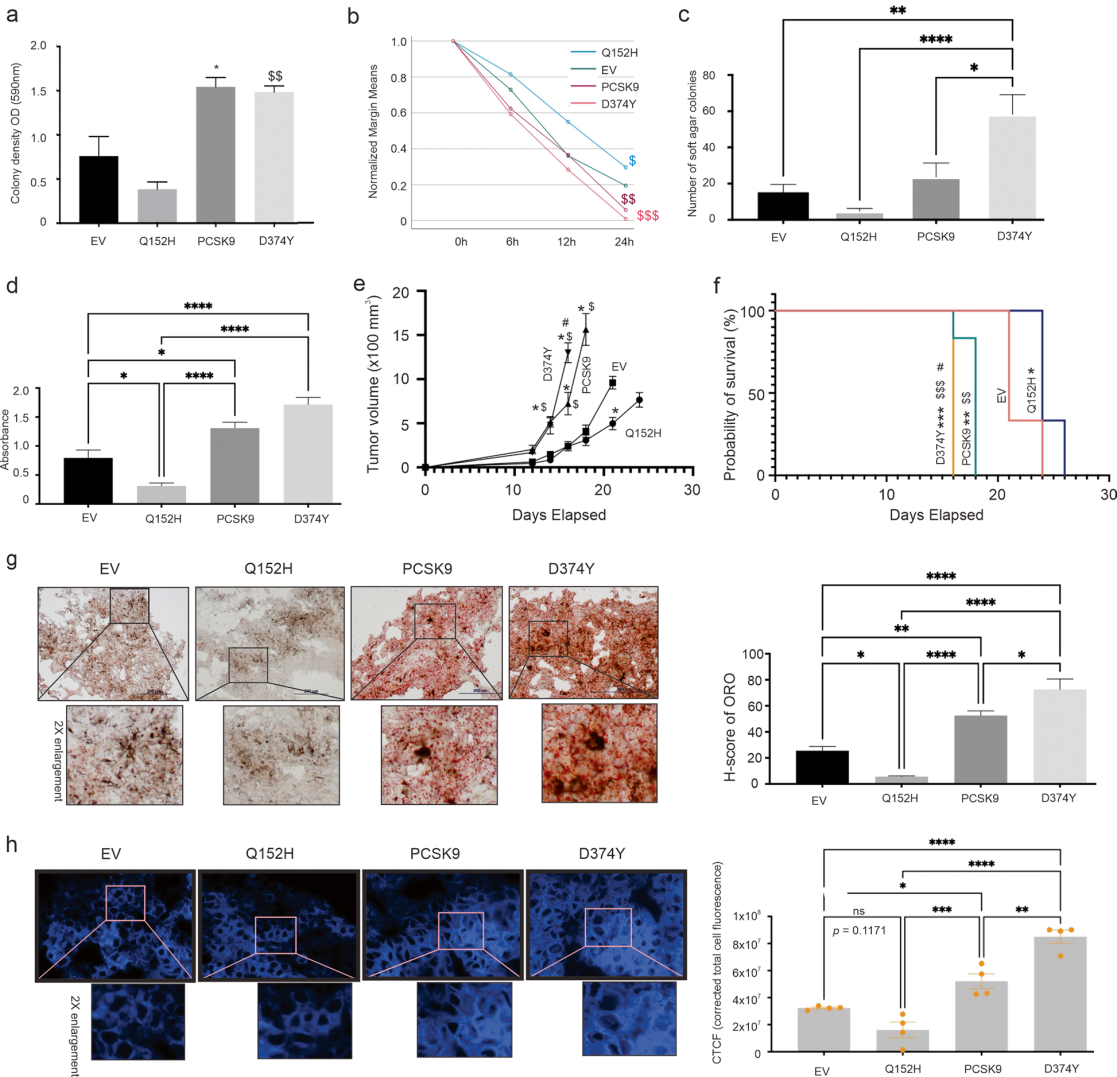
PCSK9 promotes B16 melanoma cell-derived oncogenesis. (**a**) Quantification (mean ± standard deviation/SD) of colony formation by B16 empty vector (EV), PCSK9, D374Y, and Q152H cells based on 3 repeats. See Supplementary Fig. 2b for typical images. Statistical analysis was performed with one-way ANOVA, followed by post-hoc analysis using SPSS. * *p* < 0.05 in comparison to EV; $$: *p* < 0.01 in comparison to Q152H. (**b**, **c**) Quantification of wound healing and soft agar analyses based on 3 repeats. See Supplementary Fig S2b and 2c for typical images. $ *p* < 0.05 in comparison to EV; $$: *p* < 0.05 in comparison to EV, $$$: *p* < 0.01 in comparison to EV. (**d**) Quantification (mean ± SD) of Oil Red O staining of the indicated B16 cells. Experiments were repeated 3 time; typical images are presented in Additional file 2: Fig S2f. (**e**, **f**) Allografts were produced with the indicated B16 cell lines (5 mice/line) in C57BL/6 mice. Experiments are terminated at the endpoint (tumor volume ≥ 1000 mm^3^). Tumor volumes (mean ± standard error/SE) (**e**) and survival curves are prepared (**f**). Logrank test was performed. *: *p* < 0.05 and ***: *p* < 0.001 compared to EV. $$: *p* < 0.01 and $$$: p < 0.001 in comparison to Q152H; #: *p* < 0.05 in comparison to PCSK9. (**g**, **h**) Oil Red O (ORO) (**g**) and filipin (**h**) staining of allografts produced by B16 EV (EV), PCSK9, D374Y, and Q152H cells. Five tumors per genotype were stained; typical images are presented (left panel) along with quantification (right panel). The marked regions were enlarged two-fold. *: *p* < 0.05, **: *p* < 0.01, and ****: *p* < 0.0001

Although melanomas (and many other cancer types) show the association between elevated PCSK9 expression and risk of poor OS (Fig. 1), the dominant approach utilized in PCSK9-related cancer studies was mainly via its downregulation. We have thus taken a comprehensive approach to investigate PCSK9’s role in melanoma. B16 and its derivative lines are the most common murine melanoma cells used in syngeneic mouse melanoma studies [59]. We established B16 lines stably expressing empty vector (EV), PCSK9 (wild type), GOF variant D374Y, and LOF variant Q152H (Additional file 2: Fig S2a). In comparison to B16 EV cells, B16 PCSK9 cells possess elevated abilities of colony formation, migration (wound-healing assay), and growth in soft agar; these abilities were significantly enhanced by D374Y and reduced by Q152H (Fig. 2a-c; Additional file 2: Fig S2b-d). However, ectopic PCSK9, D374Y, and Q152H did not affect B16 cell proliferation (Additional file 2: Fig S2e), suggesting PCSK9’s impacts observed above being independent of alterations in cell proliferation. On the other hand, PCSK9’s ability in modulating these processes in vitro correlates with cellular lipids accumulated (Fig. 2d; Additional file 2: Fig S2f). Furthermore, PCSK9 enhanced the growth of B16 cell-produced allografts and reduced the survival of animals bearing B16 PCSK9 tumors compared to B16 EV tumors (Fig. 2e, f). The enhancement of tumor growth and reduction in animal survival were significantly increased in B16 D374Y tumors and decreased in B16 Q152H tumors (Fig. 2e, f). The PCSK9’s oncogenic activities in vivo mirrored the magnitude of lipid accumulation in tumors, i.e. the increase in lipid accumulation in B16 PCSK9 tumors was further elevated in B16 D374Y tumors and correspondingly reduced in B16 Q152H tumors (Fig. 2g). ORO stains neutral lipids, including esterified cholesterol which the cells uptake; the upregulation observed thus suggests a role of lipid accumulation in PCSK9-derived oncogenesis. In support of this notion, the content of biologically active unesterified cholesterol [60], stained by filipin [61] was also elevated in B16 PCSK9 tumor, and further increased in D374Y and reduced in Q152H tumors (Fig. 2h).

### Main impact of PCSK9 in modulating immune reactions

**Fig. 3:**
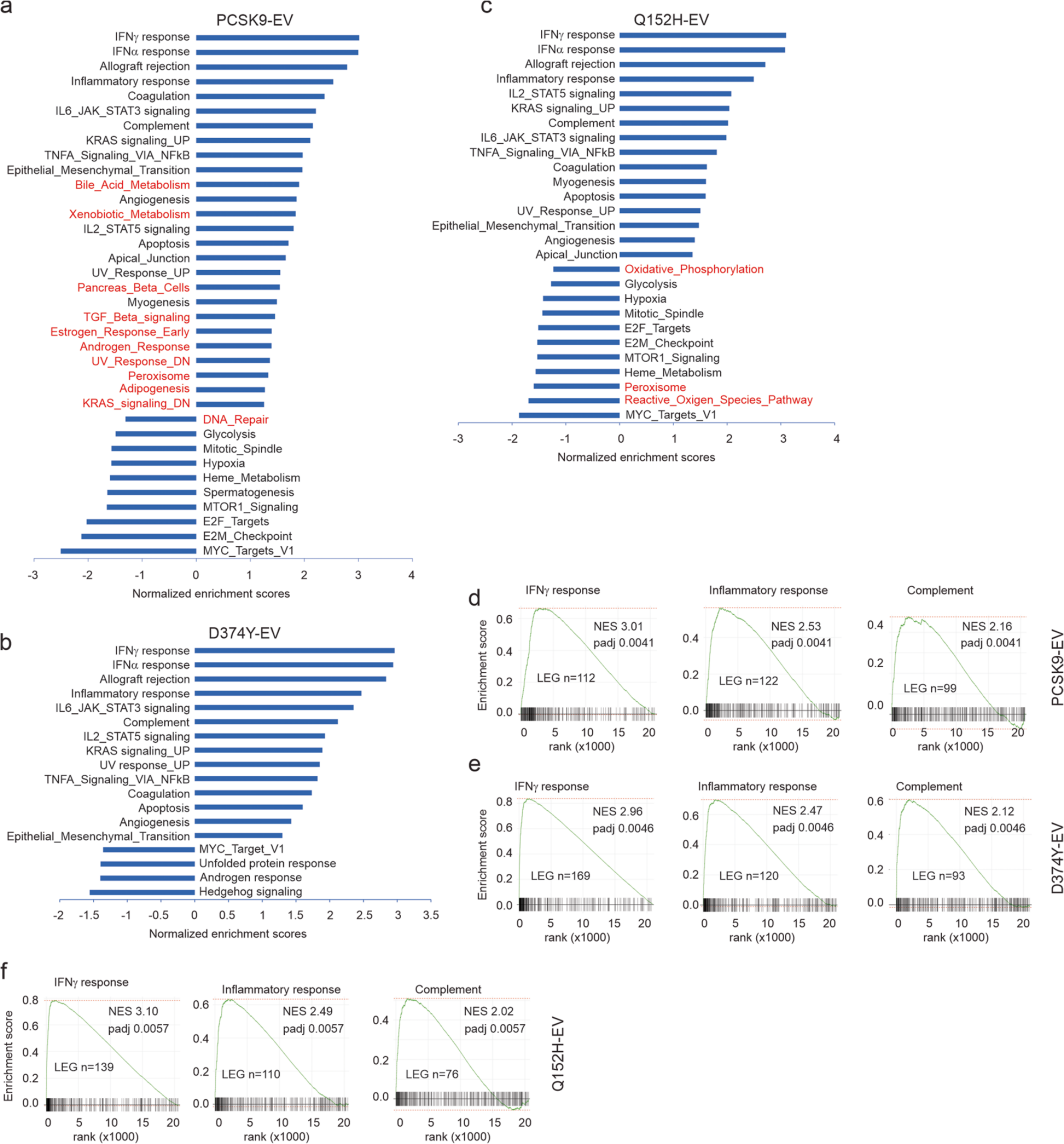
GSEA (gene set enrichment analysis) analyses on tumors produced by B16 EV, PCSK9, D374Y, and Q152H cells. (**a-c**) RNA-seq was performed on 3 tumors for each xenograft type. GSEA was carried out on MSigDB hallmark gene sets collection for the indicated comparison using Galaxy. Enriched gene sets with *p* < 0.05 are graphed according to normalized enrichment scores. The unique gene sets enriched are marked with red. (**d-f**) Top enriched gene set profiles for the indicated comparison. NES: normalized enrichment score. LEG: leading edge genes

We subsequently took a systemic approach to investigate the mechanisms underlying PCSK9-derived oncogenesis using RNA-seq. Based on our best knowledge, a similar approach has not been reported. RNA-seq was performed on 3 samples each for B16 EV, PCSK9, D374Y, and Q152H tumors. Gene Set Enrichment Analysis (GSEA) using gene expression profiles for the comparisons of PCSK9, D374Y, or Q152H to EV revealed the enrichment of 36, 18, and 27 Hallmark gene sets respectively (Fig. 3a-c; Additional file 22: Table S3a-c). All over-represented gene sets (normalized enrichment score/NES > 0) partake roles in regulating immune reactions (Fig. 3a-c). To gain more insights on these enrichments, we analyzed the top 10 enriched gene sets by considering the maximal enrichment score from the base line (enrichment score) and leading-edge genes (number and distribution), which include the enrichment of IFNγ, allograft rejection, inflammatory response, complement, and IL2_STAT5 signaling gene sets in all three comparisons: PCSK9-EV, D374Y-EV, and Q152H-EV (Additional file 22: Table S3a-c). Among the enriched gene sets of IFNγ, inflammatory response, and complement, the IFNγ gene set showed the most robust enrichment in the D374Y-EV comparison (Fig. 3d-f). Evidence thus supports a major role for PCSK9 in regulating immune reactions in melanoma, potentially independent of its function in down-regulating LDLR (D374Y or Q152H).

**Fig. 4:**
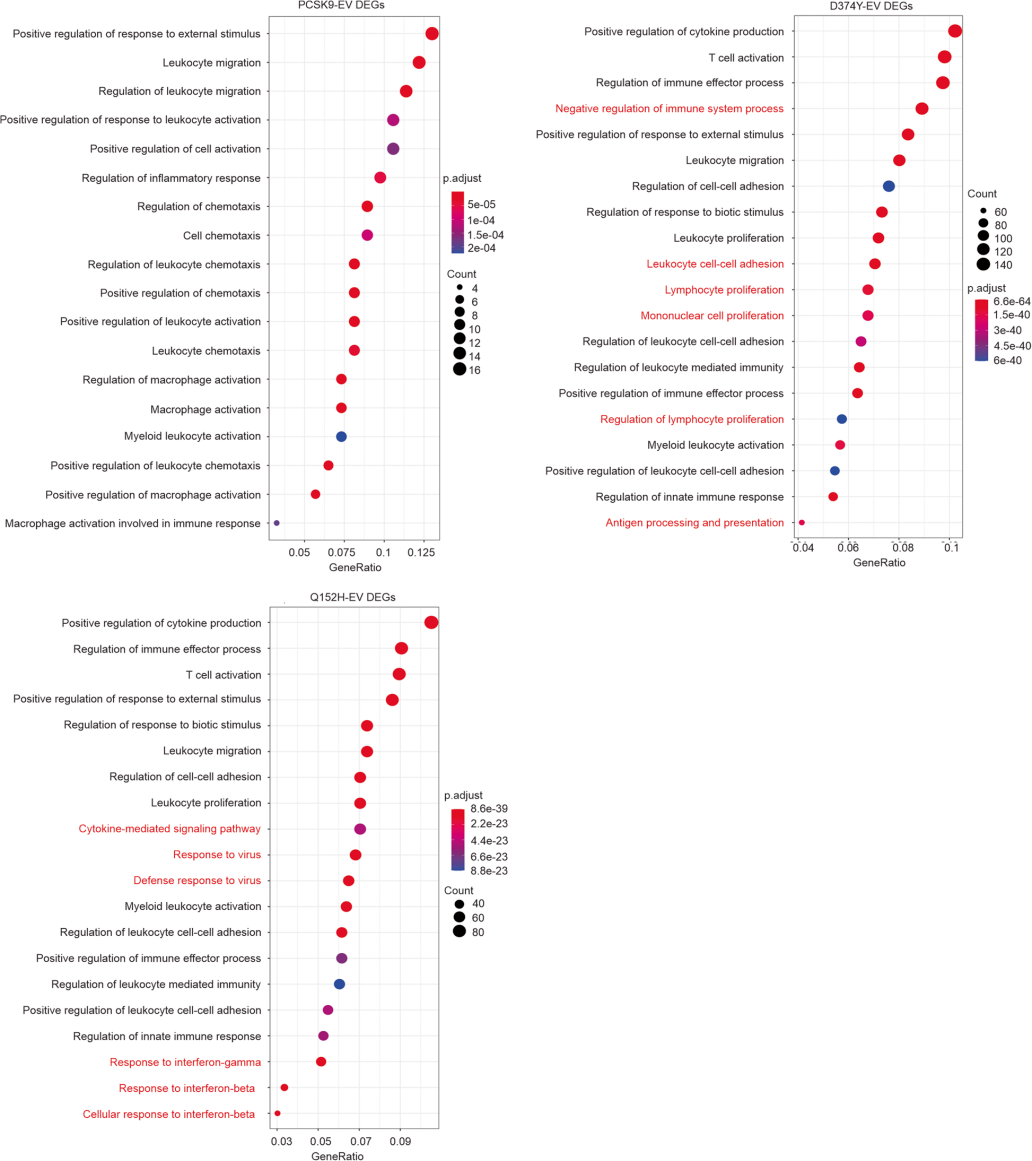
GO enrichment analysis for differentially-expressed genes (DEGs). (**a-c**) DEGs were defined as p.adj < 0.05 and fold changes ≥|1.5| in the comparisons of PCSK9 vs EV and Q152H vs EV, and fold change ≥|2| for D374Y vs EV. Over-representation of genes among these DEGs in GO events was analyzed. Top 20 enrichments are presented; unique enrichments are marked with red

Following the above analyses using rank statistics on transcriptomes, we performed GO (Gene Ontology) enrichment analysis on DEGs derived from the comparisons of PCSK9-EV, D374Y-EV, and Q152H-EV (Additional file 3: Fig S3a). These DEGs were defined as *q* < 0.05 and fold changes ≥|1.5| for PCSK9-EV and Q152H-EV or fold changes ≥|2| for D374Y-EV (Additional file 23: Table S4a-c), which reflects the elevated impact of D374Y on gene expression in B16 tumors compared to PCSK9 and Q152H. The emphasis on immune alteration in the enrichment of the three sets of DEGs was confirmed (Fig. 4). More enrichment overlaps occurred between D374Y-EV and Q152H-EV (Fig. 4). Nonetheless, unique enrichments were clearly present in individual set of DEGs and at a much higher level in D374Y-EV compared to other two settings (Fig. 4). Collectively, we demonstrated that the immune system is broadly affected by PCSK9 and that PCSK9’s activities in down-regulating LDLR are associated with unique alterations of immune reactions (Fig. 4). Intriguingly, granzyme B, granzyme C, granzyme D, perforin 1, granzyme G, and granzyme E are among the top 20 DEGs upregulated more than 64 folds (log2 > 6) in D374Y tumors compared to EV tumors (Additional file 23: Table S4b). While granzyme B, granzyme C, and perforin 1 are among the DEGs relevant to Q152H, their upregulations are at substantially lower levels (Table 1). Furthermore, D374Y tumors have increased expression of additional granzymes (Table 1).

**Table 1:** DEGs associated with immune cytotoxic activity

		**D374Y**^**i**^		**Q152H**^**i**^	
Gene	Description	log2(FC)	P-adj	log2(FC)	P-adj
Gzmb	granzyme B	7.39	4.94E-66	1.32	4.11E-08
Gzmc	granzyme C	6.95	1.76E-41	0.79	0.000571
Gzmd	granzyme D	6.72	1.49E-36		
Gzmg	granzyme G	6.36	1.80E-31		
Gzme	granzyme E	6.14	1.04E-29		
Gzmf	granzyme F	5.53	2.70E-27		
Gzma	granzyme A	4.03	2.77E-13		
Gzmk	granzyme K	2.48	0.000861		
Prf1	perforin 1	6.63	3.26E-41	1.32	8.17E-10

i: DEGs were produced in comparison to EV tumors

### Alteration of melanoma-associated immunity by PCSK9

**Fig. 5:**
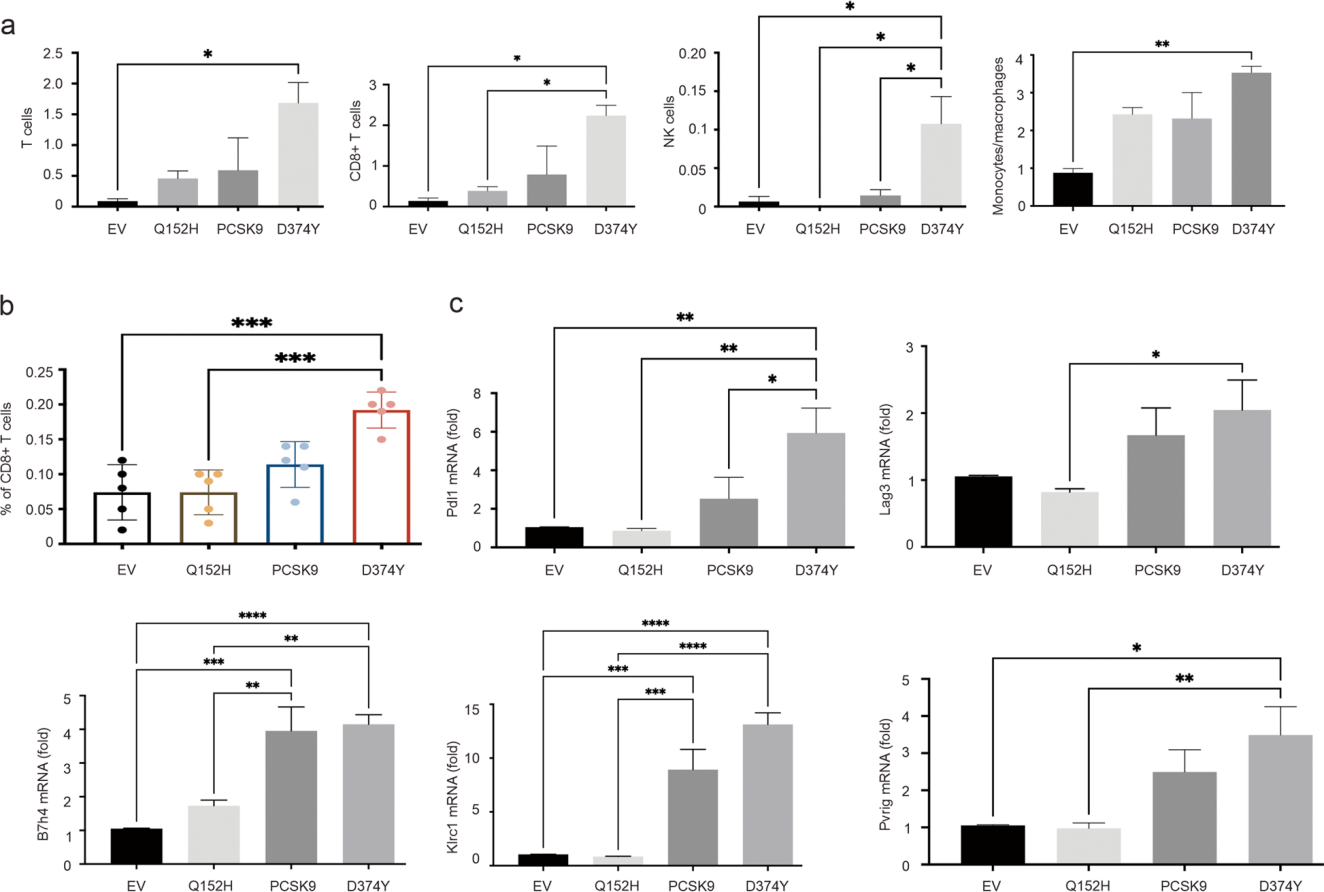
Immune cell infiltration and expression of immune checkpoints in B16 allografts. (**a**) Lymphocyte infiltration in B16EV, PCSK9, D374Y, and Q152H tumors were determined using RNA-seq expression data with mMCP computation program [52]. Means ± SDs are graphed. *: *p* < 0.05 and **: *p* < 0.01 in the indicated comparisons. (**b**) CD8 + T cell contents in B16EV, PCSK9, D374Y, and Q152H tumors were determined by immunofluorescence staining. Five tumors per tumor type were stained and quantified. Typical images were presented in Additional file 4: Fig S4. (**c**) The indicated immune checkpoints were amplified by real-time PCR in allografts generated by the indicated B16 cells. *: *p* < 0.05, **: *p* < 0.01, ***: *p* < 0.001, ****: *p* < 0.0001

The unique upregulations of granzymes and perforin 1 suggest an increase in infiltration of cytotoxic T and NK cells in B16 D374Y tumors. Estimation of tumor associated immune cells using RNA-seq data and the mMCP computation program [52] revealed significant increases in T cells, CD8 + T cells, and NK cells in D374Y tumors (Fig. 5a). An increase in CD8 + T cells in D374Y tumors was confirmed by immunofluorescence staining (Fig. 5b; Additional file 4: Fig S4). A significant increase in monocytes/macrophages also occurred in D374Y tumors (Fig. 5a), which is in accordance with more prominent upregulation of 25 MHC II loci in D374Y tumors (Table 2). Monocytes differentiate into macrophages and antigen-presenting cells (APC, dendritic cells) which express MHC II. These DEGs are clustered together in the “MHC II node” (Additional file 3: Fig S3b).

**Table 2:** DEGs of MHC II locus

		**D374Y**		**Q152H**	
Gene	Description	log2(FC)	P-adj	log2(FC)	P-adj
H2-DMa	H2^i^, class II, locus DMa	5.86	2.48E-73	2.99	2.99E-60
H2-Eb2	H2, class II antigen E beta2	5.696	2.57E-27	1.71	1.11E-12
H2-Aa	H2, class II antigen A, alpha	5.596	8.44E-43	1.07	1.02E-05
H2-Eb1	H2, class II antigen E beta	5.48	1.49E-42	1.28	2.47E-07
H2-Ab1	H2, class II antigen A, beta 1	5.23	4.31E-41	1.46	3.88E-09
H2-DMb1	H2, class II, locus Mb1	4.86	1.16E-76	1.54	3.81E-10
H2-Q7	H2, Q region locus 7	4.17	2.26E-47	1.41	3.57E-09
H2-Q6	H2, Q region locus 6	4.11	6.37E-22	1.38	4.97E-08
H2-T10	H2, T region locus 10	3.68	1.45E-52	1.16	5.98E-06
H2-M3	H2, M region locus 3	3.50	1.44E-36	2.10	4.26E-26
H2-T23	H2, T region locus 23	3.42	2.00E-65	1.53	2.17E-31
H2-K1	H2, K1, K region	3.24	6.42E-66	1.25	9.00E-08
H2-M2	H2, M region locus 2	3.02	2.32E-05		
H2-D1	H2, D region locus 1	2.94	1.26E-80	1.14	1.09E-06
H2-DMb2	H2, class II, locus Mb2	2.90	1.30E-07		
H2-T9	H2, T region locus 9	2.74	3.58E-15		
H2-Q4	H2, Q region locus 4	2.71	4.05E-81	0.89	5.03E-09
H2-T22	H2, T region locus 22	2.22	2.31E-50	0.67	6.94E-05
H2-Q10	H2, Q region locus 10	2.20	0.0003479		
H2-Q2	H2, Q region locus 2	2.12	0.0033861		
H2-Q1	H2, Q region locus 1	1.95	0.0117126		
H2-Bl	H2, blastocyst	1.71	0.0426706		
H2-Ob	H2, O region beta locus	1.65	8.58E-05		
H2-Oa	H2, O region alpha locus	1.55	0.0215398		
H2-T24	H2, T region locus 24	1.14	0.0351915		

i: histocompatibility 2

While the above evidence suggests an increase in immune activities in B16 D374Y tumors, which may reflect effort by the host defense system to counter the elevated oncogenic actions of D374Y tumors, cancers on the other hand need to dampen these immune reactions to facilitate their growth and progression. In support of this possibility, we observed upregulations of multiple immune checkpoints in D374Y tumors, including *Pdl1* (*Cd274*), *Lag3*, *B7h4*, *Klrc1 (Nkg2a)*, and *Pvrig* (Fig. 5c). The checkpoint activities of these proteins have been reported [62–66]. Additionally, 9 more murine immune checkpoints equivalent to human immune checkpoints documented in the TISIDB (tumor-immune system interactions and drug bank database) platform [55] were upregulated in D374Y tumors, including TGFβ1 (Table 3) which plays a major role in tumor immune evasion [67]. On the other hand, increases in granzyme B, granzyme C, perforin 1 and a set of MHC II locus expression also occurred in Q152H tumors (Tables 1 and 2); however, these tumors display no upregulation in immune checkpoints (Fig. 5c), implying an immunocompetent TME for Q152H tumors which may explain the Q152H-derived inhibition of tumor growth (Fig. 2e). Collectively, evidence supports that D374Y tumors are associated with a more immunosuppressive TME.

**Table 3:** Upregulations of immune checkpoints in D374Y tumors

Human Gene	Gene ID	Mouse gene	Gene ID	log2(FC)	StdErr	P.values	P.adj
CD244	51,744	Cd244a	18,106	2.500953	0.521179	1.60E-06	3.40E-05
CD274	29,126	Cd274	60,533	2.018645	0.350979	8.85E-09	2.61E-07
CD96	10,225	Cd96	84,544	3.421428	0.496495	5.53E-12	2.27E-10
CSF1R	1436	Csf1r	12,978	2.680561	0.335431	1.33E-15	7.68E-14
CTLA4	1493	Ctla4	12,477	2.340851	0.598397	9.16E-05	0.001376
IL10	3586	Il10	16,153	2.504071	0.596343	2.68E-05	0.00046
LAG3	3902	Lag3	16,768	3.234851	0.311465	2.87E-25	3.35E-23
PDCD1	5133	Pdcd1	18,566	4.632033	0.534352	4.38E-18	3.21E-16
PDCD1LG2	80,380	Pdcd1lg2	58,205	2.624019	0.398335	4.47E-11	1.70E-09
TGFB1	7040	Tgfb1	21,803	1.265117	0.310199	4.53E-05	0.000732
TIGIT	201,633	Tigit	1E + 08	3.786159	0.51301	1.58E-13	7.83E-12

### PCSK9 DEGs derived from B16 tumors predict poor prognosis of melanoma

**Fig. 6:**
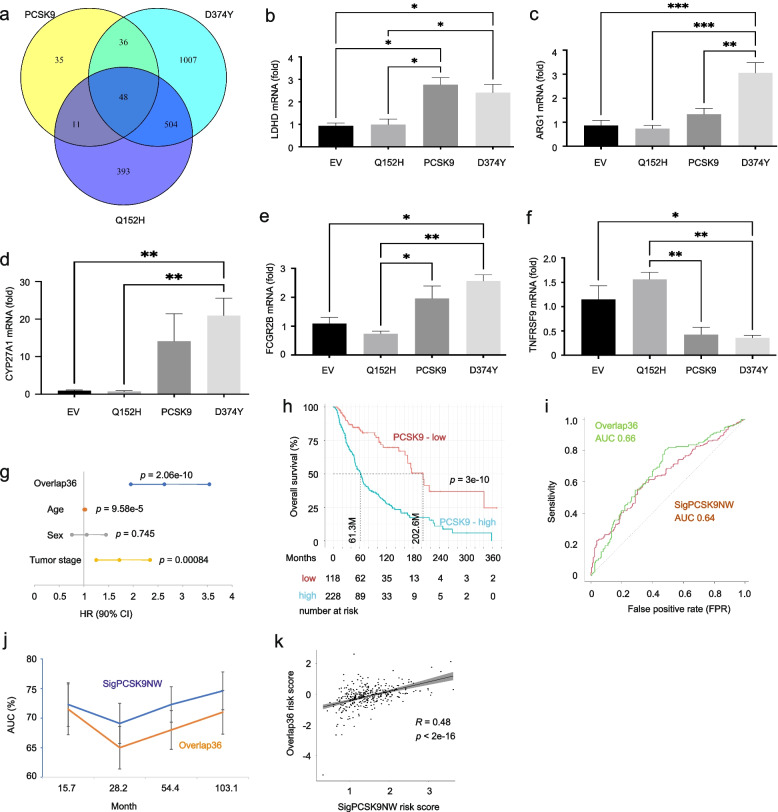
Common DEGs induced by both PCSK9 and D374Y predict poor prognosis of melanoma. (**a**) Venn Diagram of DEGs relative to PCSK9, D374Y, and Q152H tumors in comparison to B16 EV tumors. The overlapping genes between PCDK9 and D374Y DEGs are named as Overlap36. (**b-f**) Real-time PCR confirmation of the indicated component genes of Overlap36. Quantification was based on 3 repeats and was presented as fold changes relevant to EV tumors. *: *p* < 0.05, **: *p* < 0.01, and ***: *p* < 0.001 for the indicated comparisons. (**g**, **h**) Based on the TCGA PanCancer Atlas SKMC dataset, Overlap36 risk scores were calculated. The score’s potential in predicting melanoma fatality risk (**g**) and stratifying poor OS (**h**) are shown. Statistical analysis was performed using logrank test. (**i**, **j**) ROC (receiver operating characteristic) (i) and time-dependent ROC (j) curves for SigPCSK9NW and Overlap36 in discrimination of poor OS within the TCGA PanCaner Atlas SKMC dataset. (**k**) Correlation of Overlap36 risk score with SigPCSK9NW risk score in predicting poor OS of melanoma was determined using Spearman correlation in the TCGA PanCancer Atlas SKMC dataset; the correlation coefficient R and *p* value are included

The relevance of the DEGs generated from B16 allografts is evident by the pathways enriched being involved in both melanoma and tumorigenesis in large. To further analyze these DEGs, we took notice of PCSK9 and D374Y’s facilitative role and Q152H’s inhibitory role in B16 tumorigenesis (Fig. 2e, f) to suggest the presence of common factors underlying PCSK9- and D374Y-derived oncogenic actions. Of note, we observed *n* = 36 overlapping DEGs between DEGs of PCSK9-EV and D374Y-EV comparisons (Fig. 6a); these DEGs display the same change in directionality (upregulation vs downregulation) in reference to EV (Table 4). This property along with the observations that both PCSK9 and D374Y promote tumor growth (Fig. 2e) supports these genes contributing to melanoma pathogenesis. These 36 genes are enriched in a set of processes including those regulating leukocyte migration, cellular response to cytokine stimulus, protein processing, and metabolism (Additional file 5: Fig S5), which are relevant to PCSK9’s association with immune reactions observed in this study and its reported role in metabolism [68]. Among these 36 genes, *Arg1* (arginase), *Cd5l*, *Fcgr2b* (Fc receptor, IgG, low affinity IIb), *Il33* (interleukin 33), *Saa3*, *Lbp, Dpp4,* and *Tnfrsf9* (tumor necrosis factor receptor superfamily, member 9) have direct roles in the immune system (Additional file 24: Table S5a). Additionally, among these 36 DEGs, 22 of their human counterpart genes show modest correlative expressions with PCSK9 in human melanomas (*n* = 433) at *p* < 0.05 (Additional file 24: Table S5b). We further confirmed the upregulation of *Ldhd* in both D374Y and PCSK9 tumors compared to EV tumors and observed the downregulation of *Ldhd* in Q152H tumors (Fig. 6b). Upregulations of *Arg1, Cyp27a1,* and *Fcgr2b* in D374Y/or PCSK9 with their downregulation in Q152H tumors were observed (Fig. 6c-e). Consistent with RNA-seq analysis, *Tnfrsf9* was downregulated in both D374Y and PCSK9 tumors but showed increased expression in Q152H tumor (Fig. 6f). Collectively, evidence supports that the 36 PCSK9-D374Y overlap genes (Overlap36) recapitulate the key features of PCSK9 network.

**Table 4:** 36 up- and downregulated common DEGs in B16 melanoma shared by PCSK9-WT and D374Y in comparison to control

Gene	Gene name	Log2 FC	Functions in melanoma^c^	Ref
Eda2r	ectodysplasin A2 receptor	1.46^a^, 1,75^b^	*EDA2R* expression was negatively correlated with prognosis	[69]
Ldhd*	lactate dehydrogenase D	1.34, 1,45	NA	
Car3*	carbonic anhydrase 3	1.09, 3.28	NA	
Arg1*	arginase	1.09, 2.36	Enhancing activities promoting melanoma	[70]
Cnr2*	carbonyl reductase 2	1.05, 1.44	NA	
Cd5l	CD5 antigen-like	0.96, 2.99	NA	
Agap2*	ArfGAP with GTPase domain, ankyrin repeat and PH domain 2	0.95, 1.13	Identified as part of a 6-gene prognostic factor for melanoma patients, AGAP2 was highly expressed in melanoma tissues compared with normal healthy control *(p* < 0.05, Student’s t-test, *n* = 10)	[71]
Gm16316	predicted gene 16316	0.95, 1.68	NA	
Serpinb8*	serine (or cysteine) peptidase inhibitor, clade B, member 8	0.87, 2.19	NA	
Gpx3	glutathione peroxidase 3	0.87, 1.90	Inhibition of melanoma via inhibiting HIF1-α; negative association with poor prognosis in melanoma patients	[72]
Epha5	Eph receptor A5	0.84, 1.99	High EphA5 expression is associated with longer overall survival time	[73]
H19	H19, imprinted maternally expressed transcript	0.83, 1.89	Facilitating melanoma pathogenesis	[74]
Fcgr2b*	Fc receptor, IgG, low affinity IIb	0.83, 1.85	Suppressing CD8 + T cells-derived cytotoxic actions in patients with melanoma	[75]
Omg*	oligodendrocyte myelin glycoprotein	0.83, 2.22	NA	
Cryzl2	crystallin zeta like 2	0.83, 1.80	NA	
Sgk1*	serum/glucocorticoid regulated kinase 1	0.81, 1.12	Facilitating CD4 + cell differentiation via regulating IL4 and IFN-γ	[76]
Gimap7*	GTPase, IMAP family member 7	0.80, 1.56	NA	
Slco2a1	solute carrier organic anion transporter family, member 2a1	0.78, 1.53	NA	
Il33*	interleukin 33	0.78, 1.45	Likely contributing to immunosuppressive TME in metastatic melanoma	[77]
Tbc1d9*	TBC1 domain family, member 9	0.77, 1.37	NA	
Dapk1*	death associated protein kinase 1	0.76, 1.50	A pro-apoptotic factor in melanoma cells	[78]
Loxl1	lysyl oxidase-like 1	0.76, 1.62	NA	
F7	coagulation factor VII	0.73, 3.68	NA	
Gas6	growth arrest specific 6	0.72, 1.71	Facilitating activation of MAPK/ERK, PI3K/Akt and JAK/STAT	[79]
Selenop (SELP)*	selenoprotein P	0.70, 1.02	NA	
Saa3	serum amyloid A 3	0.70, 2.53	NA	
Lbp*	lipopolysaccharide binding protein	0.70, 1.97	NA	
Dpp4*	dipeptidylpeptidase 4	0.66, 1.58	Loss of DPP4 associates with melanoma progression	[80]
Cyp27a1*	cytochrome P450, family 27, subfamily a, polypeptide 1	0.60, 2.63	Cyp27a1 is a transcriptional target of melanogenesis-associated transcription factor (MITF). Cyp27a1 is necessary for the generation of a reactive metabolite that inhibits cellular proliferation in melanoma	[81]
Itga6*	integrin alpha 6	-0.63, -1.38	Decrease in metastatic SKCM tissues correlates with immune cell infiltration; Iga6 expression was also remarkably negatively correlated with overall survival in SKCM patients	[82]
Scn1a	sodium channel, voltage-gated, type I, alpha	-0.81, 1.19	NA	
Tenm4	teneurin transmembrane protein 4	-0.82, -2.50	Lower *TENM4* mRNA levels have been observed in skin cutaneous melanoma (TCGA dataset & GTEx project) compared to normal tissues	[83]
Garnl3	GTPase activating RANGAP domain-like 3	-0.93, -1.67	NA	
Slc4a4	solute carrier family 4 (anion exchanger), member 4	-0.95, -2.23	NA	
Tnfrsf9*	tumor necrosis factor receptor superfamily, member 9	-0.97, -1.61	As a costimulatory receptor in immune cells, TNFRSF9 enhances immune cell infiltration. Furthermore, elevated TNFRSF9 mRNA expression and *TNFRSF9* hypomethylation correlated with improved overall survival. In patients receiving anti-PD-1 immunotherapy, reductions of *TNFRSF9* correlated with poor response	[84]
Miga1	mitoguardin 1	-2.52, -3.71	NA	

a: PCSK9 vs EV

b: D374Y vs EV

c: for human homologue genes of mouse 36 overlapped DEGs

^*^: component genes of Overlap36sub

We analyzed Overlap36’s potential in stratification of poor OS of SKCM. We first matched 34 of the 36 murine genes to the human SKCM PanCancer Atlas dataset (for simplicity, these 34 genes are referred to as Overlap36) and merged them into a megagene (Overlap36 risk score) using coefficients generated by multivariate Cox analysis following our system [85, 86] as described in Fig. 1. The risk score predicts poor OS at HR (hazard ratio) 2.72, 95% CI (confidence interval) 2.05–3.6, and *p* = 4.44e-12; the prediction is independent of age at diagnosis, sex, and tumor stages (Fig. 6g). Overlap36 risk score effectively stratifies the risk of poor OS (Fig. 6h). In comparison to SigPCSK9NW, Overlap36 predicts the risk of poor prognosis with comparable efficiency, evident by their ROC and time-dependent ROC curves (Fig. 6i, j). Additionally, Overlap36 risk score correlates with SigPCSK9NW risk score (Fig. 6k). Considering SigPCSK9NW was derived by directly modeling the SKCM PanCancer Atlas dataset, the comparable biomarker potential observed for Overlap36 validates it as a novel and effective prognostic signature of melanoma. The novelty of Overlap36 is further supported by 20 out of 34 of its component genes that have yet to be linked to melanoma (Table 4).

### Overlap36 as a novel multigene panel in predicting response to ICB

**Fig. 7:**
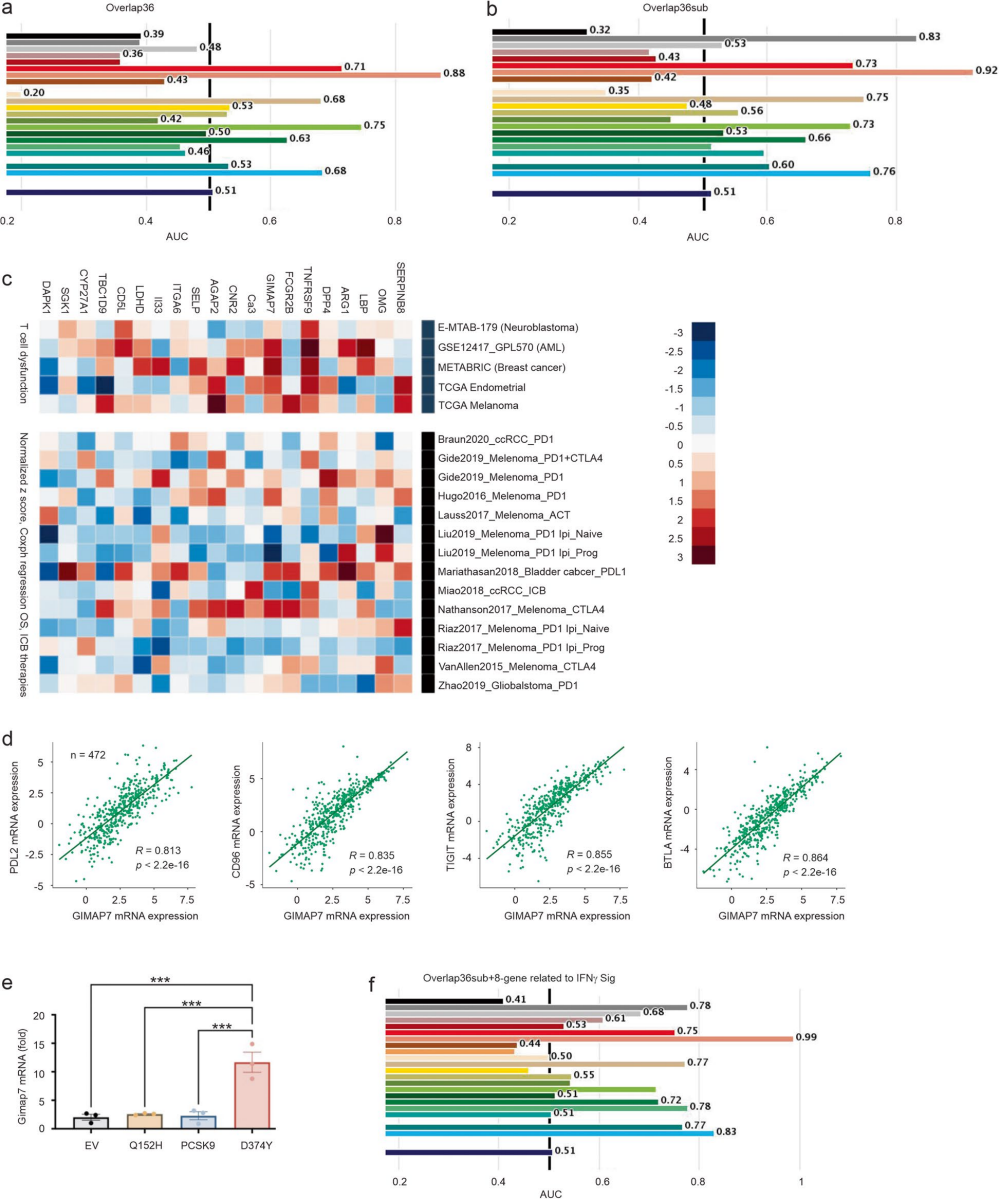
Prediction of melanoma response to ICB immunotherapy by Overlap36. (**a**, **b**) Overlap36 and Overlap36sub multigene panels were analyzed for prediction of resistance to ICB in 25 cohorts treated with ICB. The details of individual cohorts are presented in Supplementary files. (**c**) Association of Overlap36sub component genes with T cell dysfunction value (top panel) and worst survival in cancer types treated with the indicated ICB (bottom panel). Ipi: Ipilumuman anti-CTLA4; Prog: progressed; PD1.Ipi_Prog and PD1.Ipi_Naive: treatment of melanoma by anti-PD1 on tumor progressed or naïve on Ipi. (**d**) Spearman correlation of GIMAP7 with PDL2, CD96, TIGIT, and BLTA in SKMC. (**e**) Reverse real-time PCR analysis of mouse Cimap7 gene expression in the indicated tumors (*n* = 3 tumors per tumor type). Cimap7 mRNA expressions in B16 Q152H, PCSK9, and D374Y tumors were presented at fold change to its expression in B16 EV tumors. (**f**) Prediction of resistance to ICB by Overlap36sub plus the 8-gene panel conserved between D374Y DEGs and IFNγ signature [98] in melanoma and other cancer types in 25 cohorts organized by TIDE [57]

Melanoma is the most lethal skin cancer owing to its high metastatic potential. Of note, 9 component (human) genes of Overlap36 are expressed at significantly higher levels in metastatic melanoma compared to primary melanoma (Additional file 6: Fig S6). Among them, four (CAR3, CD5L, GIMAP7, and TBC1D9) are novel to melanoma (Table 4), while CD5L, FCGR2B, IL33, and TNFRSF9 have direct roles in regulating immune reactions (see their mouse homologue genes in Additional file 24: Table S5a). Since metastatic melanomas are most sensitive to ICB [12], the above observations indicate the potential of Overlap36 in evaluating melanoma responses to ICB. Additionally, SERPINB9 (Serpin Family B Member 9) was recently reported to enhance resistance to ICB [56]; SERPINB8 is a component gene of Overlap36 with unknown functions in melanoma (Table 4). By using the TIDE (tumor immune dysfunction and exclusion) program [56, 57], SERPINB8 predicts resistance to ICB in 14 of 25 cohorts at AUC values > 0.5, including NSCLC (AUC 0.68), melanoma (AUC 0.72), and gastric cancer (AUC 0.76). The slightly improved prediction observed with SERPINB8 compared with SERPINB9 (Additional file 7: Fig S7a) further supports a biomarker potential of Overlap36 in predicting response to ICB. Indeed, Overlap36 predicts ICB response (Fig. 7a) with effectiveness approaching TIDE, MSI.Score, and others (Additional file 7: Fig S7b). Considering that TIDE [56], MSI.Score (microsatellite instability) [87, 88], TMB (tumor mutational burden) [89–92], CD274 [93], CD8 [94], IFNG (IFNγ) [95], T.Clonality [96], B.Clonality [97] and Merck18 (T-cell-inflamed signature) [95] were constructed for the purpose of predicting response to ICB, and the recent FDA approval granting the use of pembrolizumab (PD1 blockade) therapy for solid tumors in adults with high MSI and TMB score [63], the matching efficacy of Overlap36 validates its potential as a novel predictor of cancer response to ICB.

**Fig. 8:**
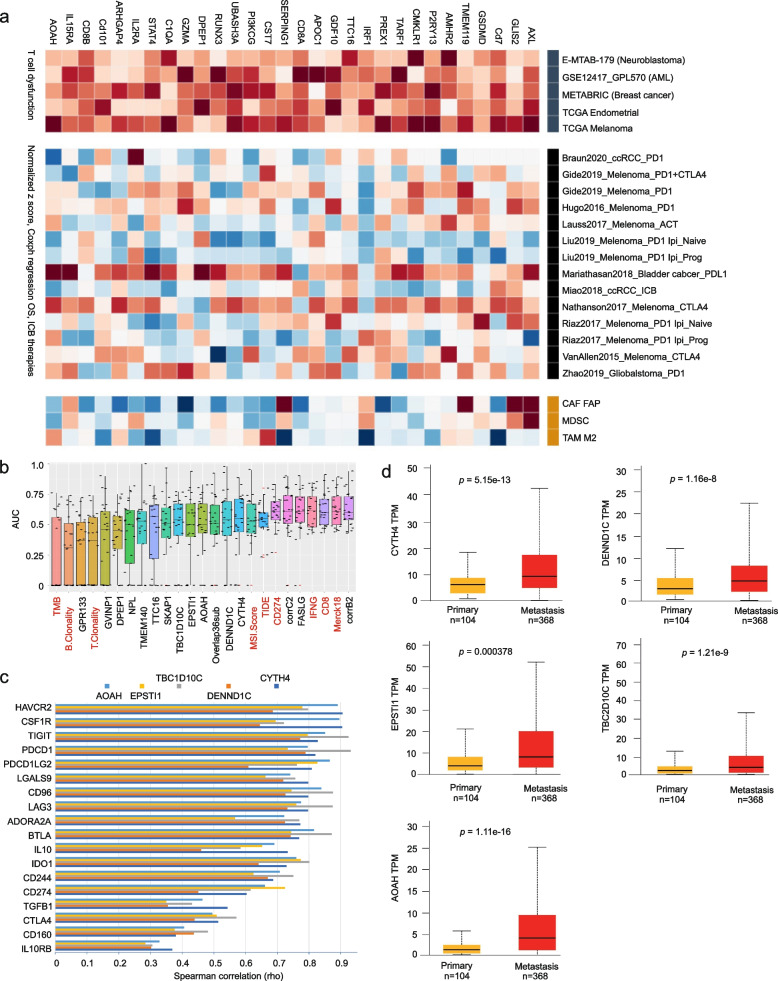
Identification and characterization of novel and effective biomarkers in assessing response to ICB. (**a**) The 30 top-ranked genes among 143 DEGs selected for their association with T cell dysfunction (Additional file 27: Table S8). CAF FAP: cancer-associated fibroblasts fibroblast activation protein; MDSC: myeloid-derived suppressor cells; TAM M2: M2 tumor-associated macrophages. (**b**) Evaluation of the indicated genes and multigene panels produced in this study along with published genes and signatures (red) for their biomarker value by AUC in discriminating resistance to ICB in 25 cancer populations treated with ICB. The evaluation was performed using the TIDE platform. (**c**) Spearman correlations between AOAH, EPSTI1, TBC1D10C, DENND1C, or CYTH4 and the indicated immune checkpoints (Y axis) in SKCM were determined using TISIDB [55]. (**d**) Analysis of the indicated gene expression in primary and metastatic melanoma using TCGA SKMC organized by UALCAN [58]

To improve the efficiency of Overlap36, we screened all component genes for their correlations with ICB response using the TIDE program [56, 57]. Numerous component genes show predictive potential for tumor immune evasion in multiple cohorts (Additional file 25: Table S6). Considering the published signatures with prediction of ICB response in less than 10 cohorts at AUC > 0.5 (TMB, T.Clonality, and B.Clonality; Additional file 25: Table S6) displayed low predictive power (Additional file 7: Fig S7b), we removed individual Overlap36 genes (marked with red; Additional file 25: Table S6) which stratify ICB response at AUC > 0.5 in less than 10 cohorts, except OMG. A subset of 20 genes (Overlap36sub) was derived, which predicts tumor immune evasion at an improved efficiency compared to Overlap36 (Fig. 7a, b). The efficiency of Overlap36sub in predicting resistance to ICB is comparable with a set of well-published biomarkers (Fig. 8b). In melanoma patients treated with CTLA4 or PD1 ICB, both Overlap36 and Overlap36sub stratify poor OS (Additional file 8: Fig S8a); among the 9 published signatures, Overlap36sub is the only one stratifying poor OS in clear cell renal cell carcinoma treated with ICB (Additional file 8: Fig S8b).

The relevance of DEGs derived from B16 D374Y allografts to melanoma is further demonstrated through examining their potential in stratifying response to ICB. IFNγ is a DEG of D374Y-EV (log2fold = 5.04, padj = 4.49e-22, Additional file 23: Table S4b) and IFNγ response is one of the top gene sets enriched (Fig. 3b). Except for DAPK1, all other component genes of Overlap36sub are associated with T cell dysfunction in multiple cancer types among neuroblastoma, acute myeloid leukemia (AML), breast cancer, endometrial carcinoma, and melanoma (Fig. 7c). SERPINB8 is correlated with high levels of T cell dysfunction in endometrial cancer and melanoma, as well as poor prognosis in melanoma, bladder cancer, and glioblastoma treated with ICB (Fig. 7c, bottom panel). Among the 20 genes of Overlap36sub, 9 are novel to melanoma, including SERPINB8 and GIMAP7 (Table 4). The latter is associated with T cell dysfunction phenotype in all five cancer types (Fig. 7c, top panel) and predicts poor prognosis in melanoma treated with ICB (Fig. 7c, bottom panel). Of note, GIMAP7 mRNA expression displays impressive correlations with immune checkpoints PDL2, CD96, TIGIT, BTLA (Fig. 7d), other immune checkpoints in melanoma, and across 30 cancer types (Additional file 9: Fig S9a). As a single gene, GIMAP7 predicts responses to ICB in 12 of 25 cohorts at AUC value > 0.5, 5 cohorts at AUC value > 0.7, and 3 cohorts at AUC value > 0.8 (Additional file 9: Fig S9b), which is significantly better than TBM, T.Clonality, and B.Clonality (Additional file 7: Fig S7b). In further support of the above analyses, we confirmed *Gimap7* upregulation in B16 D374Y allografts (Fig. 7e).

A 10-gene IFNγ signature predicts response to ICB in melanoma [98] and separates responders from non-responders to pembrolizumab (anti-PD1 antibody) in patients with melanoma [95]. Recently, signatures of TIDE were reported in multiple cancer types, including a 275-gene TIDE panel for SKCM [56]. Among the 10 component genes of IFNγ signature, 8 are present in D374Y DEGs (Additional file 26: Table S7a); among 275 genes of SKCM TIDE, 67 are present in D374Y DEGs (Additional file 26: Table S7b). Both the 8-gene and 67-gene D374Y DEG panels effectively stratify response to ICB in melanoma and other cancer types (Additional file 10: Fig S10). Addition of Overlap36sub only marginally improved the biomarker value of the 67-gene panel (data not shown), suggesting a high level of similarity between both panels. Nonetheless, addition of Overlap36sub to the 8-gene panel enhances the predictive power (Fig. 7f; comparing Fig. 7f to Fig. 7b or to Additional file 10: Fig S10). Taken together, the above analyses provide comprehensive evidence for the network affected by D374Y being highly relevant to human melanoma and displaying important biomarker values in evaluating tumor immune evasion.

### General tumor immune evasion property of D374Y DEGs as well as identification of novel and effective biomarkers of ICB response

The retainment of 8 of 10 IFNγ signature genes and 67 out of 275 SKCM TIDE genes within the D374Y DEGs indicates a general property of the D374Y network in association with immune evasion. To examine this possibility, we have matched 1283 human genes from the 1598 murine DEGs derived from B16 D374Y allografts, screened all 1283 genes for their T cell dysfunction values in 5 datasets (neuroblastoma, leukemia, breast cancer, endometrial cancer, and melanoma) organized by TIDE using the Regulator Prioritization function in the TIDE platform [56, 57], and selected these with upregulations associated with T cell dysfunction phenotypes in all 5 datasets. The resultant 143 genes consist of 11% (143/1283) of the human gene population (Additional file 27: Table S8). A predominant proportion (116/143 = 81%) of these human genes function in the immune system, including those of CD3 subunits of T-cell receptor complex (CD3E, CD3D, CD3G), CD8 molecules (CD8A, CD8B), complements (C1qa, C2), pathogen-associated molecular patterns (PAMPs) TLR8 (toll like receptor 8), chemokine (CCL4, CCL5, CCR7, CXCR6, CMKLR1, XCR1, CXCL9), and others (Additional file 27: Table S8). We confirmed the significant upregulations of mouse *Ccl4, Ccl5, Ccr7,* and *Cxcl6* in tumors produced by B16 D374Y cells (Additional file 11: Fig S11a-d). The top 30 ranked DEGs of human homologue genes by their relevance with tumor immune evasion [57] include genes functioning in the immune system, signaling (PI3KCG), and others (Fig. 8a). The discovery of AXL with established roles in promoting tumor immune evasion [99] as the top-ranked gene (Fig. 8a) provides additional evidence for D374Y DEGs or its network in shaping immunosuppressive TME in melanoma. On the other hand, while GLIS2, a top-ranked gene among the 143 genes (Fig. 8a, Additional file 27: Table S8), contributes to leukemia [100], its association with resistance to ICB and melanoma has yet to be identified. Nonetheless, such association is strongly supported by increase of GLIS2 expression in tumors with T cell dysfunction among 4 of 5 cancer populations (Fig. 8a, top panel), its association with poor prognosis in melanoma, bladder cancer, and ccRCC treated with ICB (Fig. 8a, middle panel), as well as its upregulation in stromal cells with demonstrated roles in immune evasion: CAF (cancer-associated fibroblasts) and MDSC (myeloid-derived suppressor cells) (Fig. 8a, bottom panel). These 143 candidates include 12 genes with limited knowledge for their involvement in tumor response to ICB and melanoma; TTC16 and AOAH are among the top 30-ranked genes (Fig. 8a).

These 12 genes possess promising biomarker potential in predicting response to ICB in numerous cohorts, particularly CYTH4, DENND1C, TBC1D10C, EPSTI1, and AOAH (Additional file 12: Fig S12). In comparison to 9 published biomarkers, CYTH4, DENND1C, AOAH, EPSTI1, TBC1D10C, and SKAP1 discriminate ICB resistance approaching the most effective biomarkers: Merk18, CD8, IFNG, CD274, and TIDE (Fig. 8b). Furthermore, TTC16, EPSTI1, CYTH4, NPL, AOAH, SKAP1, DENND1C, and TBC1D10C share high levels of similarities with CD8, IFNG, and Merck18 in predicting ICB resistance (Additional file 13: Fig S13); for instance, Merck18 correlates with CYTH4, NPL, AOAH, SKAP, DENND1C, and TBC1D10C at the Spearman R value of 0.7, 0.77, 0.7, 0.86, 0.78, and 0.75 respectively (Additional file 13: Fig S13), providing a strong validation for their biomarker potential in predicting ICB response. Additionally, CYTH4, DENND1C, AOAH, EPSTI1, and TBC1D10C exhibit strong correlations with a set of immune checkpoints not only in SKCM but also across a spectrum of human cancers (Fig. 8c; Additional file 14: Fig S14). In a comparison to AXL with an established role in enhancing resistance to ICB, CYTH4, DENND1C, AOAH, EPSTI1, and TBC1D10C display a stronger correlation with immune checkpoints in human cancers (Additional file 14: Fig S14). Except TGFB1, CD160, and IL10RB, correlations with other immune checkpoints by the above 5 genes are at Spearman R > 0.5 with some reaching 0.8, 0.9, and above (Fig. 8c, Additional file 15: Fig S15). CYTH4, DENND1C, AOAH, EPSTI1, and TBC1D10C expressions are upregulated in metastatic melanoma compared to primary tumors (Fig. 8d). Collectively, these 5 genes present characteristics of efficacious biomarkers in predicting resistance to ICB not only in melanoma but also across other cancer types.

As a single gene, FASLG (a gene encoding FAS ligand/FASL) possesses an impressive biomarker value in predicting resistance to ICB in all 25 cohorts organized by TIDE in a comparable efficiency to Merck18, CD8, IFNG, and CD274 (PD-L1) (Fig. 8b, Additional file 16: Fig S16a). FASLG strongly associates with multiple immune checkpoints in SKCM and multiple human cancers (Additional file 16: Fig S16b). While the FASL-FAS pathway is established for its roles in the immune system [101], the discovery of FASLG’s role as a robust biomarker of ICB therapy remains novel. In view of the novel and potent biomarker potentials of CYTH4, DENND1C, AOAH, EPSTI1, TBC1D10C, and FASLG in assessing response to ICB therapy, we took an additional effort and confirmed their murine counterpart genes being upregulated in B16 D374Y cell-produced allografts (Additional file 11: Fig S11e-j).

### Construction of two robust multigene panels as indicators of response to ICB

We further examined the biomarker potential of the 143 genes related to T cell dysfunction (Additional file 27: Table S8). Using TIDE, we derived the AUC values for all 143 genes in predicting resistance to ICB in all 25 cohorts [56, 57], and performed Spearman correlation analyses on their AUC values (Additional file 17: Fig S17). From 3 correlation regions (corrA, corrB, and corrC; Additional file 17: Fig S17), we derived two multigene panels: 6-gene panel corrB2 and 9-gene panel corrC2 (Additional file 18: Fig S18). Their biomarker values match those of the two most powerful biomarkers Merck18 and IFNG (Fig. 8b; Additional file 18: Fig S18). Furthermore, corrC2 is the only panel predicting resistance to PD1 blockade therapy at AUC 0.8 in the “Ruppin2021_PD1_NSCLC” dataset (Additional file 18: Fig S18). Collectively, evidence supports corrB2 and corrC2 as novel and effective multigene panels of ICB response.

### Tumor-derived PCSK9 as a critical source of PCSK9 in facilitating melanoma growth

**Fig. 9:**
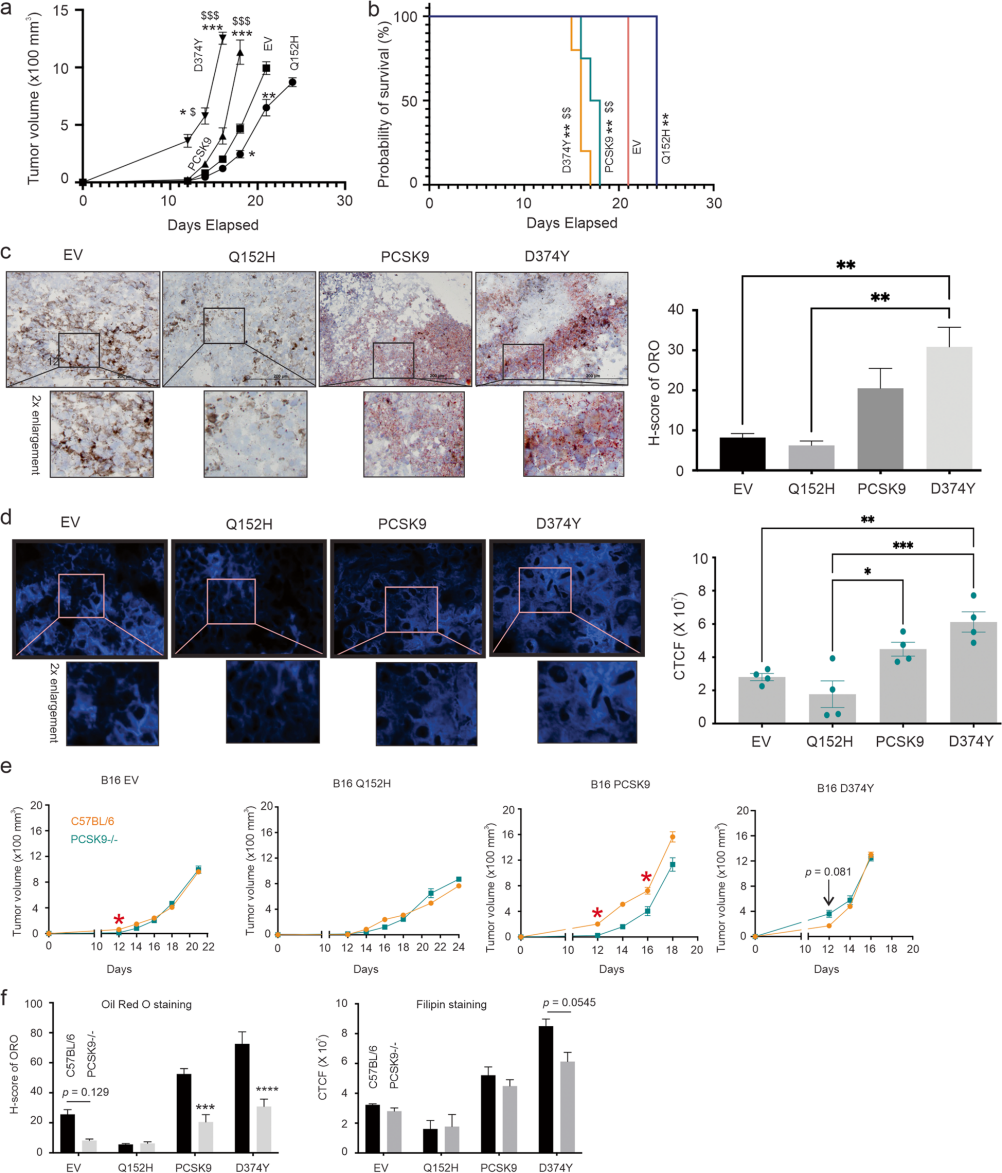
Knockout PCSK9 in mice marginally impacts melanoma growth. (**a**, **b**) B16 EV, PCSK9, D374Y, and Q152H cells were used to produce subcutaneous allografts in *Pcsk9*^*−/−*^ male mice (4 mice per cell line). Experiments were terminated at tumor volume endpoint (≥ 1000 mm^3^). Tumor volumes and survival cure were constructed. **: *p* < 0.01 compared to EV; $$: *p* < 0.001 and $$$: *p* < 0.0001 compared to Q152H. (**c**, **d**) Oil Red O (**c**) and filipin (**d**) staining of the indicated allografts (5 tumor for C57BL/6 and 4 tumors for *Pcsk9*^*−/−*^). Typical images (left panel) and quantifications (means ± SD) are graphed. **: *p* < 0.01. (**e**) Comparisons of the growth for the indicated B16 tumors in C57BL/6 and *Pcsk9*^*−/−*^ mice. *: *p* < 0.05 at the indicated time points determined by 2-tailed Student’s t-test. (**f**) Quantification of Oil Red O and filipin staining in the indicated B16 tumors produced in C57BL/6 and *Pcsk9*^*−/−*^ mice. ***: *p* < 0.001 and ****: *p* < 0.0001 in the indicated comparisons

With PCSK9 emerging as an important oncogenic factor, it presents an appealing opportunity to target PCSK9 for cancer therapy. For this application, our knowledge on PCSK9’s oncogenic involvement needs to be strengthened. The PCSK9 protein utilized by tumor can originate from circulation (host) or tumor. To delineate the impact of PCSK9 from each source, we generated allografts from B16 EV, PCSK9, D374Y, and Q152H cells in *Pcsk9*^*−/−*^ mice, and observed similar tumor growth profiles compared to C57BL/6 mice, i.e. inhibition and enhancement of tumor growth by Q152H and PCSK9 respectively, as well as further enhancement by D374Y in *Pcsk9*^*−/−*^ mice (Fig. 9a) and C57BL/6 mice (Fig. 2e). Survival curves produced in *Pcsk9*^*−/−*^ mice (Fig. 9b) and C57BL/6 mice (Fig. 2f) also showed similar trends. Like C57BL/6 mice, intratumoral accumulation of lipid was observed in D374Y and PCSK9 tumors compared to allografts produced by B16 EV and B16 Q152H cells (Fig. 9c, d). Evidence thus supports a critical oncogenic function of tumor-derived PCSK9. Nonetheless, host PCSK9 is likely involved in melanoma growth. The upregulations of *Pdl1, Lag3, B7h4, Klrc-1,* and *Pvrig* immune checkpoints observed in D374Y tumors in C57BL/6 mice (Fig. 5c) did not reach statistical significance (*p* > 0.05) in tumor produced in *Pcsk9*^*−/−*^ mice, but the trends remained (Additional file 19: Fig S19a-e). This suggests a scenario for which host PCSK9 further facilitates melanoma progression; in its absence, a larger sample size may be required to detect statistically meaningful elevations of immune checkpoint expression in melanoma. Alternatively, D374Y tumors may utilize other immune checkpoints to facilitate their growth in *Pcsk9*^*−/−*^ mice. In support of this possibility, we observed a significant elevation of *Cd96* in B16 D374Y tumors compared to both the EV and Q152H tumors produced in *Pcsk9*^*−/−*^ mice (Additional file 19: Fig S19f). As an immune checkpoint, CD96 contributes to immune escape [102].

We subsequently compared the competence of C57BL/6 and *Pcsk9*^*−/−*^ mice in B16 tumors. Among the 4 groups of allografts, the growth of B16 PCSK9 tumors was attenuated in *Pcsk9*^*−/−*^ mice, and the knockout mice did not notably reduce the growth of B16 EV, B16 D374Y, and B16 Q152H cell-derived tumors (Fig. 9e). In comparisons to tumors produced in C57BL/6 mice, PCSK9 and D374Y allografts generated in *Pcsk9*^*−/−*^ mice accumulated less esterified cholesterol (ORO staining) but not active cholesterol (filipin staining) (Fig. 9f), implying a role of tumor-derived PCSK9 in facilitating cholesterol availability to melanoma. Collectively, the above observations suggest tumor-derived PCSK9 being more critical in supporting tumor growth compared to the host source of PCSK9. This knowledge is likely important in targeting PCSK9 in cancer therapy (see Discussion for details).

## Discussion

The recent development of PCSK9 as an oncogenic factor is appealing owing to its action in regulating cholesterol homeostasis. Cholesterol is required for cell proliferation [14] and plays a major role in shaping permissive TME for cancer progression [19]. Physiologically, PCSK9 reduces cholesterol uptake by downregulation of LDLR on cell surface; this action presents a dilemma on whether PCSK9 facilitates oncogenesis via enhancing cholesterol availability. We present here a comprehensive set of indirect evidence in favor of PCSK9 facilitating melanoma at least in part via regulating lipid/cholesterol metabolism. The GOF mutant D374Y has increased binding affinity to LDLR, leading to an elevation of LDL-cholesterol in the circulation [47, 103]; D374Y enhances a set of processes relevant to the tumor formation in B16 cells in vitro and B16 cell-derived allografts in vivo along with increases in both esterified and unesterified cholesterol in comparison to wild-type PCSK9. Both oncogenic actions and cholesterol accumulation stimulated by PCSK9 and D374Y were inhibited by Q152H (Fig. 2e-h; Additional file 2: Fig S2f), a dominant negative variant that reduces the secretion of endogenous PCSK9 by retaining the protein in the endoplasmic reticulum [48], and hence impedes its action in downregulating LDLR [104]. Additionally, CYP27A1 is upregulated by both PCSK9 and D374Y but not Q152H (Fig. 6d; Table 4); CYP27A1 plays a major role in maintaining cholesterol homeostasis [105]. The molecular details responsible for PCSK9 to facilitate melanoma in the utilization of cholesterol need further investigations. Cholesterol uptake under PCSK9 upregulation might be mediated by LDLR and scavenger receptors; in B16-derived cells, PCSK9 is unable to downregulate cell surface LDLR [41] and scavenger receptors are known to uptake cholesterol in cancer cells [106]. However, we cannot exclude the possibility that increases in cholesterol accumulation were a result of the elevations in tumor growth stimulated by PCSK9. The enhanced lipid accumulation occurs at least partially by tumor cells, as it occurs in B16 cells in vitro (Additional file 2: Fig S2f). Nonetheless, lipid accumulation can also take place in TME in vivo.

The connection to TME is in line with the roles of cholesterol within TME in shaping immunosuppressive microenvironment [19]. Intriguingly, AXL is the top ranked DEG associated with T cell dysfunction in multiple cancer types (Fig. 8a). AXL (a member of the Tyro3-Axl-Mer/TAM family) is a receptor tyrosine kinase and is activated by binding to its ligand GAS6 (growth arrest specific 6); the GAS6/AXL pathway promotes tumor progression in part by facilitating immune evasion [99]. GAS6 is a common DEG related to PCSK9 and D374Y tumors (Table 4). It is thus tempting to propose that the actions of the GAS6/AXL signaling contribute to PCSK9-initiated immunosuppressive TME in melanoma.

The dominant aspect of PCSK9 in promoting melanoma is its systemic impact on the immune system. While PCSK9 has been elegantly demonstrated to facilitate immune evasion by downregulation of MHC I from the cancer cell surface [43], our research reveals a systemic influence of PCSK9 on the immune system that is likely beyond its impact on MHC I at least in melanoma. In this regard, B2M (beta-2 microglobulin) [107], a critical subunit of the MHC I complex, is a D374Y DEG (log2Fold 2.74, padj = 2.93e-13, Additional file 23: Table S4b). The alterations in the immune system caused by PCSK9 occur on CD8 molecules, factors critical for CD8 + T cell functions (granzymes and perforin 1), CD3 subunits of T-cell receptor complex (CD3E, CD3D, CD3G), complements, PAMP (TLR8/toll like receptor 8), cytokines (TGFβ1 and INFγ), chemokine (CCL5, CCR7, CCL4, CXCR6, and others), PI3KCG [signaling regulating immune checkpoint (PD-L1) expression in cancer cells [108]], and a set of immune checkpoints (PD1, PD-L1, PD-L2, CTLA4, TIGIT, CD96, LAG3, and others) (Tables 1, 2, 3 and 4; Additional file 27: Table S8; Fig. 8c; Additional file 9: Fig S9; Additional file 14: Fig S14).

The systemic impact of PCSK9 on the immune system sets a stage for our discoveries of novel biomarkers of ICB therapy. CYTH4, AOAH, DENND1C, TBC1D10C, EPSTI1, SERPINB8, and GIMAP7 are novel and robust individual genes in predicting resistance to ICB in multiple cancer types (Fig. 8; Additional file 7: Fig S7; Additional file 9: Fig S9; Additional file 12: Fig S12). These genes have no known involvement in melanoma and tumor immune evasion. CYTH4 (cytohesin 4) is a candidate of guanine nucleotide-exchange factor (GEF) for ARF1 and ARF5 [109]. AOAH (acyloxyacyl hydrolase) hydrolyzes acyloxyacyl-linked fatty acyl chains from bacterial lipopolysaccharides [110]. DENND1C (DENN domain containing 1C) is a GEF activating Rab35 that regulates actin cytoskeleton [111]. TBC1D10C (TBC domain family member 10C) is a Ras GTPase-activating protein (GAP) and an inhibitor of Ras and calcineurin [112]; its role in regulating macrophage’s cytoskeleton [113] supports its association with immune escape observed in this study. TBC1D9 (another member of the family and a potential GAP) is a component gene of Overlap36 (Table 4), indicating the importance of this family in melanoma immune evasion. Similarly, SERPINB8 (serpin family B member 8) is a member of the family of serine protease inhibitors [114], and a set of its related family members were present within the D374Y DEGs, including Serpina3f, Serpina3g, Serpinb9, Serping1, Serpinb6b, Serpina3n, Serpina3c, and Serpina3h (Additional file 23: Table S4b). Human SERPINB9 has been reported to associate with resistance to ICB [56]. GIMAP7 (GTPase, IMAP family member 7) is a GTPase in the GIMAP family [115]; Gimap3 and Gimap1 are also present in the list of D374Y DEGs (Additional file 23: Table S4b). EPSTI1 (epithelial stromal interaction 1) promotes B cell activation via NFκB signaling [116]. In addition to melanoma, CYTH4, AOAH, DENND1C, TBC1D10C, EPSTI1, SERPINB8, and GIMAP7 stratify responders and non-responders to ICB in other cancer types (Additional file 7: Fig S7; Additional file 9: Fig S9; Additional file 12: Fig S12). This potential is supported by their impressive correlation with numerous immune checkpoints across 30 human cancers (Additional file 9: Fig S9; Additional file 14: Fig S14). Additionally, we observed *Faslg* upregulation in D374Y tumor (Additional file 11: Fig S11j). Human FASLG represented one of the most robust biomarkers identified to date in predicting resistance to ICB, including TIDE, IFNG, CD8, and Merck18 (Fig. 8b; Additional file 16: Fig S16). The status of melanoma-derived FASL in induction of lymphocyte apoptosis and its contribution to immune escapes remains controversial [117–120]. While whether FASL mediates immune escape via activation of FAS in lymphocytes remains unclear, our observation for FASL’s capacity in predicting resistance to ICB not only in multiple cohorts and cancer types but at a comparable robustness with established high profile biomarkers (TIDE, IFNG, CD8, and Merck18) [56, 94, 95] strongly supports its potential to assess response following ICB therapy.

This research yielded 3 multigene panels: Overlap36sub, corrB2, and corrC2. The latter two panels consist of genes with direct functions in the immune system (Additional file 18: Fig S18). GVINP1 is a long non-coding RNA (lncRNA) and a pseudogene induced by interferons with unclear function [121]. It is thus intriguing that GVINP1 predicts resistance to ICB therapy in melanoma and other cancer as a single gene and a component gene of corrB2 (Fig. 8b; Additional file 18: Fig S18). Both corrB2 and corrC2 are novel and highly effective indicators of response to ICB (Fig. 8b; Additional file 18: Fig S18). Overlap36sub was derived from DEGs shared by PCSK9 and D374Y tumors, which highlights the relevance of PCSK9 network derived from B16 tumors to melanoma progression. Furthermore, while not all component genes of Overlap36sub directly regulate the immune system (Table 4), the panel affects pathways relevant to immune reactions (Additional file 5: Fig S5), significantly predicts resistance to ICB (Fig. 8b), and supports the concept of PCSK9 contributing to immunosuppressive TME via affecting cholesterol metabolism. Nonetheless, the biomarker potentials of our discoveries require further validations, particularly in view of the small datasets within TIDE.

The systemic impacts of PCSK9 on the immune system observed here support the ongoing efforts to target PCSK9 alone and/or in combination with ICB in cancer therapy. Nonetheless, our study suggests the focus should be on tumor-derived PCSK9 instead of PCSK9 in circulation, as tumor-produced PCSK9 appears to be much more critical than the host or circulating PCSK9 in facilitating melanoma. This possibility is consistent with Q152H inhibiting melanoma growth compared to EV, particularly in *Pcsk9*^*−/−*^ mice (Fig. 9a, b), which might be attributable to the LOF mutant’s ability to suppress endogenous wild-type PCSK9. Our research thus calls for caution in targeting PCSK9 in the circulation with anti-PCSK9 antibodies and PCSK9 vaccine in cancer patients. Rather, these approaches might be effective in cancer prevention, which may shed light on why vaccination of mice for PCSK9 followed by cancer cell implantation was marginally impactful against melanoma and breast cancer growth [32, 40]. The lack of reductions in cancer incidence in clinical trials on cardiovascular conditions by anti-PCSK9 antibodies evolocumab and alirocumab [45, 46] might be attributable to the presence of cancerous lesions at time when the antibodies were administrated.

## Conclusions

We report here multiple major discoveries. 1) The relevance of PCSK9’s role in maintaining cholesterol homeostasis to its oncogenic actions remains unknown. We provide the first evidence for PCSK9 facilitating melanoma pathogenesis via enhancing intratumoral cholesterol accumulation. 2) PCSK9 enhances melanoma via systemic alterations of the immune system, which is consistent with PCSK9-mediated elevations of intratumoral cholesterol accumulation. 3) We identified 7 novel biomarkers of ICB therapy, CYTH4, DENND1C, AOAH, TBC1D10C, EPSTI1, GIMAP7, and FASLG. These genes not only effectively predict responses to ICB therapies but also positively correlate with multiple checkpoint expressions in melanoma and across 30 human cancer types; the correlations were observed at impressively high levels, with highest at Spearman correlation 0.932. 4) We have constructed 3 novel and powerful multigene panels predicting responses to ICB treatment. 5) Tumor-derived PCSK9 is more critical in supporting melanoma oncogenesis compared to circulation PCSK9. This knowledge is essential in developing PCSK9-based cancer therapy.

## Supplementary Information

**Additional file 1:** **Figure S1.** Construction of SigPCSK9NW in estimation of fatality risk of melanoma.

**Additional file 2:** **Figure S2.** PCSK9 reduces B16 cell migration and growth in soft agar. (a) Stable expression of empty vector (EV), PCSK9, D374Y and Q152H mutants in B16 cells was analyzed using Western blot. Stable lines were produced using retrovirus. (b) B16 EV, PCSK9, D374Y, and Q152H cells were seeded at the indicated density and cultured for a week, followed by crystal violet staining. Experiments were repeated 3 times; typical images from a single repeat are shown. (c) Wounds (gaps) were produced in the indicated B16 cell monolayer; floating cells were removed; and wound closure was monitored as indicated. Experiments were repeated 3 times. Typical images from a single repeat are included. (d) 10^4^ cells for the indicated B16 lines were seeded in soft agar (60 mm plate). Experiments were repeated 3 times. Typical images from a single repeat are shown. (e) B16 EV, B16 PCSK9, B16 D374Y, and B16 Q152H cells were seeded at 105/well in a 6-well plate. Cell numbers were counted at the indicated time. Experiments were repeated 3 times; means ± SD are graphed. (f) Oil Red O staining of the indicated B16 monolayer cells. The marker regions are enlarged two folds.

**Additional file 3:** **Figure S3.** Characterization of DEGs relevant to PCSK9. (a) RNA-seq data for B16 EV, PCSK9, D374Y, and Q152H tumors (3 tumors per genotype) was used to generate DEGs using DESeq2 within the Galaxy platform (https://usegalaxy.org/). Heatmaps for the indicated DEGs are shown. (b) Network enrichment for D374Y DEGs. The network consists of 4 domains: MHC protein complex, receptor complex containing CD3 subunits (Cd3e and Cd3g) of T-cell receptor complex, membrane rafts and membrane microdomain, and collagen-containing extracellular matrix.

**Additional file 4:** **Figure S4.** Increases of CD8+ T cells in B16 D374Y cell-produced tumors. Tumors produced from the indicated B16 cells were immunofluorescence stained with anti-CD8 Ab and counter stained with DAPI. Five tumors per group were stained. Typical images are shown.

**Additional file 5:** **Figure S5.** Enrichment analysis for Overlap36 DEGs.

**Additional file 6:** **Figure S6.** Upregulations of Overlap36 component genes in melanoma metastasis.

**Additional file 7: Figure S7.** Prediction of resistance to ICB by SERPINB8 and Overlap36.

**Additional file 8:** **Figure S8.** Stratification of worst OS in melanoma and other cancer types treated with ICB by Overlap36 and Overlap36sub.

**Additional file 9:** **Figure S9.** Association of GIMAP7 with response to ICB.

**Additional file 10:** **Figure S10.** Prediction of cancer response to ICB by two sets of D374Y DEGs which are included in IFNγ signature (8-gene) and melanoma TIDE panel (67-gene). The TIDE signature is also included for comparison purpose.

**Additional file 11:** **Figure S11.** Real-time PCR confirmation of selected DEGs produced in B16 D374Y tumors compared to B16 EV tumors.

**Additional file 12:** **Figure S12.** Estimation of response to ICB therapy by the indicated genes using TIDE. These genes are D374Y DEGs with associations with T cell dysfunction (see Supplementary Table S7 for details).

**Additional file 13:** **Figure S13.** Correlations of novel genes showing association with T cell dysfunction with 9 published biomarkers (marked with red) of ICB.

**Additional file 14:** **Figure S14.** Spearman correlations for the indicated genes with 24 immune checkpoint proteins across 30 human cancer types.

**Additional file 15:** **Figure S15.** Spearman correlations of CYTH4 and TBC1D10C expression with the indicated immune checkpoints. Analyses were carried out with the TCGA SKCM dataset organized by TISIDB.

**Additional file 16:** **Figure S16.** FASLG is a novel indicator of response to ICB. (a) FASLG and the indicated biomarkers were analyzed for prediction of resistance to ICB in 25 cohorts organized by TIDE. (b) Association of FASLG with immune checkpoints in SKCM and other human cancers were evaluated using TISIDB.

**Additional file 17:** **Figure S17.** Correlation analysis of 143 genes for predicting response to ICB.

**Additional file 18:** **Figure S18.** CorrB2 and corrC2 effectively predict resistance to ICB. Predictions of ICB response by the indicated biomarkers were performed using TIDE. The component genes for corrB2 and corrC2 are included.

**Additional file 19:** **Figure S19.** Upregulation of immune checkpoint expression in B16 D374Y tumors produced in *Pcsk9*^*-/-*^ mice.

**Additional file 20:** **Table S1.** Sequence of primers used for analyzing mouse genes by RT-PCR.

**Additional file 21:** **Table S2.** PCSK9 associated DEGs in primary melanoma.

**Additional file 22:** **Table S3.**

**Additional file 23:** **Table S4.** (a) DEGs produced in the comparison of B16 PCSK9 tumors to B16 EV tumors. (b) DEGs produced in the comparison of B16 D374Y tumors to B16 EV tumors. (c) DEGs produced in the comparison of B16 Q152H tumors to B16 EV tumors.

**Additional file 24:** **Table S5.** (a) GO biological processes of Overlap36 genes. (b) Correlation of Overlap36 genes with PCSK9 expression in SKCM tumors (n = 433) within the TCGA PanCancer Atlas dataset.

**Additional file 25:** **Table S6.** Evaluation of the biomarker values of Overlap36 component genes in predicting tumor immune evasion.

**Additional file 26:** **Table S7.** (a) IFNgamma signature genes included in the D374Y-EV DEGs. (b) SKCM TIDE genes present the D374Y-EV DEGs.

**Additional file 27:** **Table S8.** D374Y DEGs (n = 143) associated with T cell dysfunction.

## Data Availability

All data generated and materials used in this manuscript are available upon request.

## Abbreviations

CI: Confidence interval
DEGs: Differentially-expressed genes
EV: Empty vector
GOF: Gain-of-function
GSEA: Gene set enrichment analysis
HR: Hazard ratio
ICB: Immune checkpoint blockade
LDLR: Low-density lipoprotein receptor
LOF: Loss-of-function
NW: Network
PR: Precision-recall
ROC AUC: Receiver-operating characteristic area under the curve
SKCM: Skin cutaneous melanoma
TME: Tumor microenvironment
TAM: Tumor-associated macrophages
